# A Toxicological Evaluation of Methylliberine (Dynamine®)

**DOI:** 10.1155/2019/4981420

**Published:** 2019-10-27

**Authors:** Timothy S. Murbach, Róbert Glávits, John R. Endres, Amy E. Clewell, Gábor Hirka, Adél Vértesi, Erzsébet Béres, Ilona Pasics Szakonyiné

**Affiliations:** ^1^AIBMR Life Sciences, Inc., 2800 East Madison Street, Suite 202, Seattle, WA 98112, USA; ^2^Toxi-Coop Zrt., Magyar Jakobinusok tere 4/B, H-1122 Budapest, Hungary

## Abstract

Methylliberine (CAS 51168-26-4), a methoxiuric acid, is a caffeine metabolite present at low levels in various *Coffea* plants; however, very little has been published regarding this compound and we could find no toxicological data in the public domain. Therefore, we undertook the toxicological investigation of a pure, synthetic form of methylliberine in order to evaluate its potential health hazards as a food ingredient. A (1) bacterial reverse mutation test, (2) in vitro mammalian chromosomal aberration test, (3) in vivo mammalian micronucleus test, and (4) 90-day repeated-dose oral toxicity study in rats with a 28-day recovery period were conducted. No in vitro mutagenic or clastogenic activity was observed in the presence or absence of metabolic activation up to the maximum OECD recommended test concentrations. No genotoxicity was observed in the mammalian micronucleus study up to the highest dose tested of 700 mg/kg bw. In the 90-day study, methylliberine was administered to Han:WIST rats at doses of 0, 75, 112, 150, 187, and 225 mg/kg bw/day. No mortality or morbidity was observed and no toxicologically relevant clinical effects or effects on clinical pathology parameters were observed. In male animals, test item-related effects on body weight and sexual organs, which were not reversible after a 28-day recovery period without treatment, were observed in the high-dose group. Body weight development was also slightly and reversibly depressed in the 187 mg/kg bw/day male group. No toxicological effects were observed in females. The NOAEL for females was determined to be 225 mg/kg bw/day, the highest dose tested, while the NOAEL for males was determined to be 150 mg/kg bw/day. Future studies are encouraged to corroborate the safety, and assess efficacy, of methylliberine in humans.

## 1. Introduction

**Figure 1:**
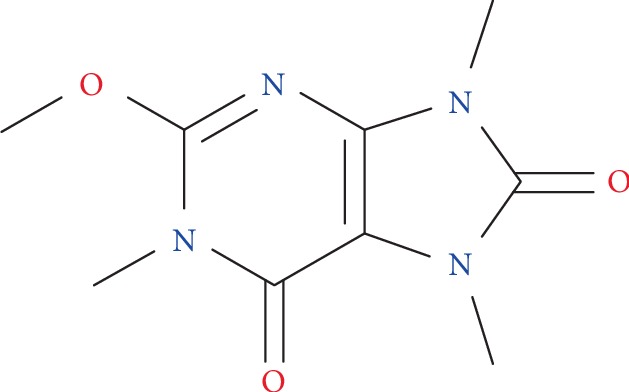
Methylliberine.

Methylliberine (CAS RN 51168-26-4; *O*(2),1,7,9-tetramethylurate) is a purine alkaloid of the methoxiuric acid group having the molecular formula C_9_H_12_N_4_O_3_ and a molecular weight of 224.22 g/mol. The structural formula of methylliberine is shown in Figure 1. In general, methoxiuric acids are metabolic derivatives of methylxanthines (e.g., caffeine, theobromine). Specifically, methylliberine is found at low levels in plants of the genus *Coffea* and is a metabolite of caffeine, via a theacrine intermediate, that is likely further metabolized to liberine as an end product [1].

Due to structural similarities to the methylxanthine caffeine, methylliberine is hypothesized to have similar physiological properties but without undesirable stimulant effects, as has previously been demonstrated for the related methoxiurate, theacrine [2–5]. The slight structural differences between theacrine and methylliberine have been hypothesized to result in slight differences in the pharmacokinetic profiles of the two compounds, with methylliberine thought to have a shorter time to reach maximum plasma concentration and a shorter half-life with respect to theacrine. Because of these hypothetical properties, there is interest in methylliberine as an ingredient in functional foods and dietary supplements, and clinical trials to investigate all three hypotheses are currently underway. Nonetheless, as of this writing there is a dearth of published information, including toxicological data, relating to methylliberine. In the current work we conducted a battery of toxicological investigations in order to evaluate the potential of methylliberine to cause genetic toxicity and possible health hazards (including major toxic effects, target organs, and the possibility of accumulation) likely to arise from repeated exposure of methylliberine and to estimate a no-observed-adverse-effect level (NOAEL) in rats.

## 2. Materials and Methods

### 2.1. Test Item

The test item was Dynamine® (Compound Solutions, Inc., Carlsbad, California, USA), a ≥98.0% pure methylliberine. Lot number 49-KY20171201 (99.6% purity) was used to carry out the studies. The test item is produced according to current Good Manufacturing Practice via a synthetic process and meets food grade specifications for identity, purity, impurities, physical characteristics, heavy metals, and microbial growth.

The studies herein described were conducted in compliance with OECD Principles of Good Laboratory Practice (GLP) [6], except for the following deviation: analytical control of the test item formulations for homogeneity and stability was not performed because no appropriate test method has been validated. We have described the OECD methods and laboratory standard operating procedures (SOP) used in previous works [7–12], and summarize them again, in brief, below.

### 2.2. Animal Husbandry

Animal acclimatization, housing, and environmental conditions for the micronucleus test in mice and the 90-day oral toxicity study in rats were in accordance with the respective OECD test guidelines applied [13, 14]. Feed (ssniff® SM R/M-Z+H complete diet for rats and mice, ssniff Spezialdiäten GmbH, Soest, Germany) and potable tap water were provided *ad libitum*. Both animal studies were conducted according to SOPs for the care and use of animals under a protocol approved by the Institutional Animal Care and Use Committee of Toxi-Coop Zrt. Additionally, the 90-day study was conducted in compliance with the National Research Council Guide for Care and Use of Laboratory Animals [15] and the principles of the Hungarian Act 2011 CLVIII (modification of Hungarian Act 1998 XXVIII) regulating animal protection.

### 2.3. Bacterial Reverse Mutation Test

Based on preliminary solubility and concentration range finding tests, dimethyl sulfoxide (DMSO) (Merck KGaA, Darmstadt, Germany) was selected as the vehicle for the test item, and test item concentrations of 5000, 1600, 500, 160, 50, 16, and 5 *µ*g/plate were employed in the main bacterial reverse mutation tests (the high-concentration was the maximum recommended concentration for soluble, noncytotoxic substances). The GLP main tests were comprised of an initial plate incorporation procedure and a confirmatory preincubation procedure conducted in triplicate according to OECD Guideline for Testing of Chemicals 471 [16] using procedures described by Ames et al. [17], Maron and Ames [18], Kier et al. [19], Venitt and Parry [20], and Mortelmans and Zeiger [21] and the SOPs of the laboratory in order to evaluate the mutagenic potential of the test item.

Mutagenicity was assessed in bacterial tester strains *Salmonella typhimurium* TA98, TA100, TA1535, and TA1537 and *Escherichia coli* WP2 *uvrA* (Moltox, Inc., Boone, NC, USA) with and without metabolic activation. Metabolic activation was provided using S9-mix prepared in the laboratory with post mitochondrial supernatant (S9) prepared from livers of phenobarbital/*β*-naphthoflavone-induced rats (Moltox, Inc., Boone, NC, USA). The sensitivity, reliability, and promutagen activation potential of the S9 was certified by the supplier using known controls and further investigated by the testing laboratory. Positive controls were 4-nitro-1,2-phenylenediamine (NPD), sodium azide (SAZ), and 9-aminoacridine (9AA) obtained from Merck Life Science GmbH (Eppelheim, Germany) and methyl methanesulfonate (MMS) and 2-aminoanthracene (2AA) obtained from Sigma-Aldrich Co. (Saint Louis, MO, USA). 2AA was the positive control with metabolic activation for all tester strains. Without metabolic activation positive controls were: NPD (TA98), SAZ (TA100 & TA1535), 9AA (TA1537), and MMS (WP2 *uvrA).* DMSO was the vehicle/negative control for NPD, 9AA, and 2AA. Ultrapure water (ASTM Type 1, prepared in the laboratory by Direct-Q5 system, Millipore) was the vehicle/negative control for SAZ and MMS. The test solutions, positive control solutions, and the S9-mix were freshly prepared at the beginning of each experiment.

Colony numbers were determined by manually counting; from this mean values, standard deviations, and mutation rates were calculated. According to the established criteria of the laboratory, the test item was considered mutagenic if:

(i) A concentration-related increase in revertant colonies occurred; AND/OR.
(ii) A reproducible biologically relevant positive response for at least one dose group occurred in at least one strain with or without metabolic activation.

An increase was considered biologically relevant if:

- The number of reversions in strains *S. typhimurium *TA98 and/or TA100 and/or *E. coli *WP2 *uvrA* was at least twofold greater than the reversion rate of the negative vehicle-control AND/OR.
- The number of reversions in strains *S. typhimurium *TA1535 and/or TA1537 was at least threefold greater than the reversion rate of the negative vehicle-control.

The test item was considered nonmutagenic if the criteria for a mutagenic response were not observed.

### 2.4. In Vitro Mammalian Chromosomal Aberration Test

The test item was dissolved in Dulbecco's Modified Eagle's (DME) medium (Sigma Aldrich, Schnelldorf, Germany), based on the results of a preliminary solubility study, and two independent experiments were conducted, in duplicate, utilizing V79 male Chinese hamster lung cells (European Collection of Authenticated Cell Cultures; Salisbury, England) grown in supplemented DME medium as the test system, in order to evaluate the clastogenic potential of methylliberine. Experiment A was conducted without and with S9-mix at treatment/sampling intervals of 3/20 hours while experiment B was conducted at treatment/sampling intervals of 20/20 and 20/28 hours without S9-mix and 3/28 hours with S9-mix. Based on preliminary cytotoxicity testing, test concentrations of 250, 500, 1000, and 2000 *µ*g/mL were utilized for the 3 hours treatments without and with metabolic activation and test concentrations of 125, 250, and 500 *µ*g/mL were utilized for the 20 and 28 hours treatments without metabolic activation.

The experiments were conducted and evaluated according to OECD Guideline for Testing of Chemicals 473 [22] using procedures described by Preston et al. [23] and Brusick [24] and the SOPs of the laboratory. DME was the negative/vehicle-control, and the positive controls (obtained from Sigma Aldrich, Schnelldorf, Germany) were ethyl methanesulfonate (EMS) without metabolic activation and cyclophosphamide monohydrate with metabolic activation. The suitability of DME as the vehicle and of EMS as the positive control without S9-mix was confirmed by the laboratory's historical database; EMS is also a known and widely used mutagen and clastogen according to the referred literature. The test solutions, positive control solutions, and the S9-mix were freshly prepared directly prior to the treatment of the cells.

### 2.5. In Vivo Mammalian Micronucleus Test

The test item was suspended in 1% methylcellulose (Sigma Aldrich, Schnelldorf, Germany) and administered twice, at 24 hours intervals, by gavage to groups of five specific pathogen-free (SPF) male Crl:NMRI BR mice (Toxi-Coop, Budapest, Hungary) at doses of 0, 175, 350, and 700 mg/kg bw. The mice weighed 32.5–36.3 g at the start of treatment. Dose and sex selection were based on the results of a preliminary toxicity test conducted in two mice of each sex at 500, 1000, and 2000 mg/kg bw. The test item solutions were freshly prepared and administered within 2 hours at a constant dose volume of 20 mL/kg bw. The positive control was cyclophosphamide (Sigma-Aldrich, Schnelldorf, Germany) dissolved in sterile water (NATURLAND Kft., Budapest, Hungary) and was administered to an additional group of five mice once at a dose of 60 mg/kg bw by intraperitoneal injection at a volume of 10 mL/kg bw.

Immediately following administration and at regular intervals until sacrifice 24 hours following the final treatment, the mice were observed for adverse reactions. Bone marrow sampling, from the exposed femurs of each mouse, was performed immediately following sacrifice by cervical dislocation. Two microscope slides per mouse (one coded for blind examination) were prepared in order to evaluate the potential of methylliberine for genetic toxicity. The study was conducted and evaluated according to OECD Guideline for Testing of Chemicals 474 [13] using procedures described by Salamone and Heddle [25].

### 2.6. 90-Day Repeated-Dose Oral Toxicity Studies in Rats

A control and five dose groups of 46–50-day-old male (171–205 g) and female (124–150 g) SPF Han:WIST rats (Toxi-Coop, Budapest, Hungary) were administered methylliberine suspended in 1% methylcellulose (Molar Chemicals Kft., Halásztelek, Hungary) for 90 (males) and 91 (females) consecutive days in general accordance with OECD test guideline 408 [14]. One hundred forty rats were randomized by weight stratification into six groups of 10 rats/sex/group that were administered the test item by gavage at doses of 0 (vehicle-control), 75, 112, 150, 187, and 225 mg/kg bw/day and two additional groups of five rats/sex that were administered the control and high-dose for further evaluation following a 28-day recovery period after the last day of treatment. All test solutions were freshly prepared each day and administered by gavage at a constant dose volume of 10 mL/kg bw within 4 hours.

Dose selection was based on the results of an unpublished 14-day dose range-finding study conducted according to OECD test guideline 407 in which groups of rats received 0 (1% methylcellulose vehicle-control), 55, 110, and 220 mg/kg bw/day for 14 consecutive days (note these dose levels were based on the micronucleus study in mice reported herein). As no adverse effects were observed, the high-dose for the 90-day study was set at 225 mg/kg bw/day.

Observations, measurements, and evaluations were conducted according to OECD test guideline 408 and SOPs of the laboratory for mortality, daily cage-side and weekly detailed clinical observations, functional observations, ophthalmologic evaluations, body weight, and food consumption (including calculation of body weight gain and feed efficiency), clinical pathology (hematology, including coagulation, and clinical chemistry), gross pathology and organ weights (including calculation of organ weights relative to body weight and brain weight), and full histopathological examinations of all control and high-dose group animals as well as histopathological examination of all gross pathological lesions observed at necropsy. Tissues and organs of the lower dose groups were also preserved in case high-dose findings warranted extended evaluation.

Subdued lighting was maintained during administration of mydriatic eye drops (Cicloplegicedol® (10 mg/mL) Laboratório Edol—Produtos Farmacêuticos S.A., Linda-a-Velha, Portugal) for ophthalmoscopy and for the remainder of each examination day. The functional observation battery (FOB), carried out during the final week of treatment, was conducted utilizing a modification of the method of Irwin [26]. On the days of sacrifice, following approximately 16 hours of food deprivation, animals were placed under deep narcosis induced with Isofluran CP® anesthesia (Medicus Partner Kft, Biatorbágy, Hungary), and blood samples were obtained from the retro orbital venous plexus. Blood sampling was not feasible in one female animal at 187 mg/kg bw/day due to over anesthesia. Therefore, clinical pathology examinations were not conducted for this animal. Animals were exsanguinated from the abdominal aorta following blood collection and subjected to necropsy.

### 2.7. Statistical Analyses

Statistical analyses were performed with SPSS PC+ software, version 4 (SPSS, Inc., Chicago, IL, USA), and Microsoft Excel version 2016 (Microsoft, Hungary) was used to check the chromosomal aberration and micronucleus tests' data for linear trends. A *p*-value of <0.05 was considered statistically significant in all tests.

#### 2.7.1. Bacterial Reverse Mutation Test

Because biological relevance was the criterion applied for the interpretation of results, no statistical evaluation was conducted.

#### 2.7.2. In Vitro Mammalian Chromosomal Aberration Test

A chi-squaretest was used to evaluate the number of aberrations (with and without gaps) and the number of cells with aberrations (with and without gaps). The number of aberrations in the treatment and positive control groups was compared to the concurrent negative control. The concurrent negative and positive controls and the treatment groups were also compared to the laboratory historical controls. The data were checked for a linear trend in number of cells with aberrations (without gaps) with treatment dose using the adequate regression analysis.

#### 2.7.3. In Vivo Mammalian Micronucleus Test

Kruskal–Wallis nonparametric one-way analysis of variance (ANOVA) was used to compare frequencies of micronucleated polychromatic erythrocytes (MPCE) in the test item and positive control groups to the concurrent negative control group and historical negative control values. The data were checked for a linear trend in mutant frequency with treatment dose using the adequate regression analysis.

#### 2.7.4. 90-Day Repeated-Dose Oral Toxicity Study in Rats

Male and female data for body weight, body weight gain, food consumption, feed efficiency, clinical pathology, and absolute and relative organ weights were evaluated separately. Heterogeneity of variance between groups was checked with Bartlett's homogeneity of variance test. If statistically significant heterogeneity was not detected, a one-way ANOVA was carried out, and positive results were further evaluated using Duncan's Multiple Range test to assess the significance of inter-group differences. Data were examined for normality using the Kolmogorov–Smirnov test if Bartlett's test was statistically significant, and nonnormal distributions were further evaluated using Kruskal–Wallis nonparametric one-way ANOVA. Inter-group comparisons were performed post hoc using the Mann–Whitney *U*-test if nonparametric ANOVA results were statistically significant. For recovery period data, homogeneity of variance between groups was assessed with an *F*-test. A pooled or separate variance estimate was obtained by performing a two-sample *t*-test depending on the result.

Statistical analyses were not performed for nonquantitative study parameters. Instead, frequencies of occurrence by sex and dose were calculated for clinical and functional observations, ophthalmoscopy, and gross and histopathological findings.

## 3. Results and Discussion

### 3.1. Bacterial Reverse Mutation Test

Summary results of the initial and confirmatory mutation tests are shown in Tables 1 and 2, respectively. No concentration-related or biologically relevant increases in revertant colony numbers were observed in any of the five tester stains at any concentration of methylliberine, either in the presence or absence of metabolic activation, in either of the performed tests. Additionally, the test item did not have inhibitory effects on bacterial growth and background lawn development was unaffected.

### 3.2. In Vitro Mammalian Chromosomal Aberration Test

#### 3.2.1. Preliminary Cytotoxicity Test

The test item did not exhibit cytotoxic activity (assessed as the Relative Increase in Cell Counts (RICC)) to V79 cells with short-term treatment, either in the presence or absence of S9-mix, up to the highest recommend test concentration (2000 *µ*g/mL) for soluble substances. Under long-term treatment without S9-mix, methylliberine exhibited approximately 100 and 75% cytotoxicity (calculated as 100-RICC) at 2000 and 1000 *µ*g/mL, respectively, while at 500 *µ*g/mL observed cytotoxicity was approximately 53%. Therefore, 2000 and 500 *µ*g/mL were chosen as the high concentrations for the short- and long-term treatments, respectively, in the main experiments.

#### 3.2.2. Main Chromosomal Aberration Experiments

The results of the main chromosomal aberration experiments are summarized in Table 3. No excessive cytotoxicity was observed in any of the tested conditions of the main experiments A and B. No concentration-related increases or statistically significant increases in the mean number of cells with chromosomal aberrations compared to concurrent or historical negative controls were observed, either with or without metabolic activation, at any test concentration under any of the tested conditions of experiments A or B. Additionally, no polyploid or endoreduplicated metaphases were observed in either experiment.

In experiment A with metabolic activation, the mean value (6 aberrant cells excluding gaps/150 cells) for the concurrent negative control was slightly above the historical control range (2–5 aberrant cells excluding gaps/150 cells). As the deviation was slight and control charts indicated the test system was under control, the value was deemed acceptable and did not influence the quality or integrity of the experiment. Also in experiment A, the mean values at several treatment concentrations were slightly above the 95% control limits of the historical data but remained within the historical control ranges as follows: at 1000 *µ*g/mL without metabolic activation and 250 and 1000 *µ*g/mL with metabolic activation, the mean values were 5 aberrant cells excluding gaps/150 cells (see Table 3); the historical control ranges both with and without metabolic activation were 2–5 aberrant cells excluding gaps/150 cells, and the upper 95% control limits were 4.11 and 4.35 aberrant cells excluding gaps/150 cells without and with metabolic activation, respectively. As there were no statistically significant differences with respect to the concurrent or historical negative controls, these deviations were considered to have occurred without biological relevance.

### 3.3. In Vivo Mammalian Micronucleus Test

#### 3.3.1. Preliminary Toxicity Test

All animals of the 2000 mg/kg dose group died within 3 h following the first treatment. Clinical signs of moderate to heavy degree, observed in all animals of each dose group, were decreased activity, narrow palpebra, hunchback posture, piloerection, and increased respiration rate. Prone position (heavy degree) was also observed in the 1000 and 2000 mg/kg groups. The clinical signs appeared within 10 minutes after dosing and persisted for five hours in the surviving animals. The same signs were observed again, in the surviving animals, following the second treatment. Based on the observed toxicity, the high-dose for the main test was set at 700 mg/kg bw and the mid- and low-doses were set at 350 and 175 mg/kg bw, respectively. No sex differences were observed.

#### 3.3.2. Main Micronucleus Test

No deaths occurred in any dose group, and no abnormal clinical signs were noted in the low dose group or negative and positive control groups. Within 30 minutes after each treatment, and persisting for four hours, moderately decreased activity was observed in the mid-dose group. Decreased activity, narrow palpebra, hunchback posture, piloerection, and increased respiration rate were observed in heavy degree in the high-dose group within 30 minutes, and persisted five hours, following both treatments. Additionally, prone position was observed between three and five hours after each treatment.

No dose-related, biologically, or statistically significant increases in frequency of MPCEs were observed in bone marrow of treated mice at 24 h after the second treatment compared to concurrent negative controls up to the highest dose tested; however, at the low-dose group, the result was statistically significant compared to the historical negative control. As this result remained within the 95% control limit of the historical negative control, it was not considered biologically relevant. There were no statistically significant differences in the ratio of immature among total erythrocytes in the treated groups compared to the concurrent negative controls; however, a slight decrease in the ratio compared to the negative control group at the high-dose group was considered evidence of bone marrow exposure. Results of the micronucleus test are summarized in Table 4.

Similar results were reported by Endres et al., in which the related methoxiurate, theacrine did not induce in vivo chromosomal damage in the bone marrow of treated mice at two consecutive doses up to 325 mg/kg bw [27]. This is in contrast to earlier reports of in vitro chromosomal aberrations induced by theacrine. Kihlman and Odmark observed induction of chromosomal aberrations during G_2_ (but not during S or G_1_ phases) in *Vicia faba *roots co-treated with 5 × 10^−2^ M theacrine and ^3^H radiolabeled thymidine [28]. Later, Kihlman reported the induction of chromosomal aberrations in Chinese hamster cells by both theacrine and caffeine as well as the induction of mutations in tryptophan-deficient *E. coli* by both substances [29]. As reported in Sections 3.1 and 3.2 of the current work, methylliberine did not induce gene mutations in bacteria or in vitro chromosomal aberrations in mammalian V79 cells when evaluated in compliance with GLP and accordance with OECD guidelines.

### 3.4. 90-Day Repeated-Dose Oral Toxicity Study in Rats

#### 3.4.1. Mortality, Clinical Observations, and Ophthalmology

No mortality occurred during the study treatment or recovery periods. A scar was observed transiently on the neck and then shoulder of a single male control group animal. Salivation before and after treatment was observed in one female each of the 187 and 225 mg/kg bw/day groups on study Days 32–48 and 32–49, respectively, and slight salivation was observed in one 225 mg/kg bw/day male during the last nine days of treatment (days 81–89). While it was considered related to the treatment procedure, because of the low incidence it was not considered related to the test item. No other clinical signs were present in any animals during general daily or detailed weekly clinical observations, and no abnormal behavior or reactions to different types of stimuli were observed during the FOB. No eye alterations were observed during the ophthalmologic examinations.

#### 3.4.2. Body Weights and Food Consumption

**Figure 2:**
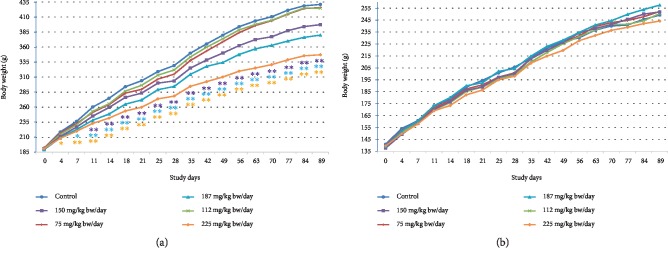
Body weights in the 90-day study. (a) Male body weights. (b) Female body weights. ^∗^*p* < 0.05, ^∗∗^*p* < 0.01 (Duncan's multiple range test).

**Figure 3:**
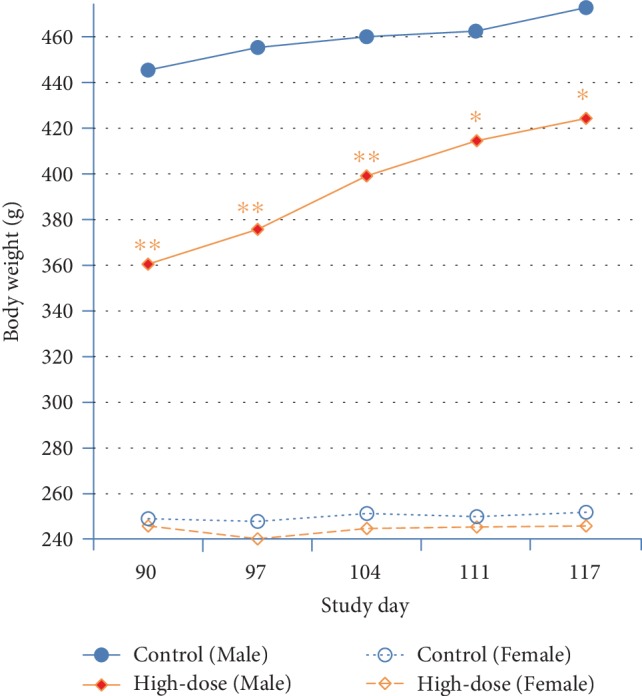
Recovery group body weights during the 90-day study recovery period. ^∗^*p* < 0.05, ^∗∗^*p* < 0.01 (*T*-test versus control).

Mean body weight was statistically significantly, dose-dependently reduced compared to controls in the 150, 187, and 225 mg/kg bw/day males throughout the entire treatment period beginning on study days 11, 7, and 4, respectively (see Figure 2(a)). The reduced body weights correlated with transient statistically significant reductions in food consumption (see [Supplementary-material supplementary-material-1] in the Supplementary Material), feed efficiency (see [Supplementary-material supplementary-material-1] in the Supplementary Material), and body weight gain with an overall statistically significant, dose-dependent decrease in body weight gain (see Table 5). The depressed body weight development was slight (<10% compared to controls) in the 150 mg/kg bw/day group and, therefore, was not considered toxicologically relevant. The difference with respect to the control in the body weight of male animals at 225 mg/kg bw/day decreased by the end of the recovery period but was not fully reversible (see Figure 3).

Body weights were comparable to controls throughout the treatment and recovery periods in the treated female groups (see Figures 2(b) and 3). A few transient differences compared to controls in body weight gain, food consumption, and feed efficiency in the treated female groups were of slight magnitude and had no impact on overall body weight development (see Table 5 and Tables [Supplementary-material supplementary-material-1] and [Supplementary-material supplementary-material-1] in the Supplementary Material).

Similar statistically significant, dose-related effects have been observed on body weight development of male rats in toxicological investigations involving theacrine [30] and methylxanthines caffeine and theobromine [31, 32] suggesting this may be a sex-specific effect of methylxanthines and their metabolites at high doses. Although, with respect to the methylxanthine, theophylline, effects on body weight have been less consistent with some studies showing reduced weight in both males and females and other showing increased body weight compared to controls in females and no effects in males [33].

#### 3.4.3. Clinical Pathology

*Hematology. *The results of the hematological evaluations in male and female rats are summarized in Table 6. In main group males, statistically significant increases in neutrophil percent (NEU), decreases in lymphocyte percent (LYM), and increased (150 mg/kg group) or decreased (187 and 225 mg/kg groups) eosinophil percent (EOS) compared to the control did not correlate with any changes in white blood cell counts (WBC). In the main group females, a statistically significant increase and decrease in NEU and LYM, respectively, with respect to controls was observed only in the high-dose group while statistically significant decreases in EOS were observed in all treated groups compared to control and a statistically significant, dose-related increase in WBC was observed in the top three dose groups compared to controls. No significant changes in WBC or percent differentials persisted in the recovery group males or females, and the observed effects in the main groups remained (with the exception EOS in the top three female dose groups) well within the historical control ranges of the laboratory and no correlating histopathology was observed; therefore, these changes were not considered toxicologically relevant.

In main group males, statistically significant dose-related increases in hemoglobin (HGB), hematocrit (HCT), mean corpuscular volume (MCV), mean cell hemoglobin (MCH), and percentage of reticulocytes (RET) compared to controls were observed. In the main group females, sporadic, statistically significant increases were observed compared to control in MCV, and sporadic, statistically significant decreases were observed compared to control in mean cell hemoglobin concentration, while RET was statistically significantly increased in all treated females compared to control although a dose-response was not clearly observed. At the end of the recovery period, the changes in MCV, MCH, and RET persisted in the high-dose male recovery group while HGB and HCT in high-dose recovery males and RET in high-dose recovery females were comparable to the respective recovery controls. Erythrocyte count was unaffected in all male and female groups compared to corresponding controls and no correlating histopathology was observed. Additionally, the magnitudes of change were minimal with respect to the corresponding controls with the majority of values remaining within or marginal to the historical control range. Thus, the observed effects on HGB and HCT in males, RET in both sexes, and various red cell indices among the sexes were considered to have occurred without toxicological relevance.

Prothrombin time was statistically significantly reduced compared to controls in the main 112 and 150 mg/kg male groups while activated partial prothrombin time was statistically significantly increased in the 187 and 225 mg/kg males and the 75 and 112 mg/kg females. These changes were sporadic, remained within historical control limits, lacked correlating histopathology, and were not present in the recovery high-dose animals following 28 days without treatment; therefore, they were not considered to have toxicological relevance.

1. *Clinical Chemistry* Dose-related statistically significant increases with respect to corresponding controls occurred in the main treatment groups for alanine aminotransferase (ALT) and albumin (ALB) in 187 and 225 mg/kg males and cholesterol (CHOL) in all male groups. Dose-related statistically significant decreases with respect to corresponding controls occurred in the main treatment groups for aspartate aminotransferase (AST) and creatinine (CREA) in 187 and 225 mg/kg females, alkaline phosphatase (ALP) in all male groups, sodium (Na^+^) in 187 and 225 mg/kg males, and chloride (Cl^−^) in all male and female groups. A dose response was less clear in the statistically significant changes observed for glucose (GLUC), inorganic phosphate (Pi), and potassium (K^+^) in males and calcium (Ca^++^) and Na^+^ in females while statistically significant changes in AST, CREA, and Ca^++^ in males; total bilirubin (TBIL), GLUC, CHOL, and Pi in females; and albumin/globulin ratio in both sexes appeared sporadic. Except for the elevations in CHOL all changes remained within or marginal to the historical control ranges of the laboratory and all high-dose recovery group values were comparable to the corresponding controls at the end of the recovery period. While dose-related, decreases in AST, ALP, and CREA are not biologically significant in the absence of correlating histopathology, dose-related increases in ALT and ALB were considered adaptive responses of the liver and/or kidneys to altered demand as no correlating histopathology was observed to suggest otherwise, and they were fully recovered after 28 days with no treatment. The elevations in CHOL, while above the historical control range, were, nonetheless, small in magnitude and fully recovered after 28 days with no treatment and, therefore, were also considered related to an adaptive hepatic response. All other changes were considered sporadic, or possibly adaptive, due to their small magnitudes, the absence of correlating histopathology, and their recovery following 28 days without treatment. The results of the clinical chemistry evaluations are shown in Table 7.

#### 3.4.4. Gross Pathology

Smaller than normal testes were observed in the main male 225 mg/kg bw/day group. This finding persisted in high-dose males following the 28-day recovery period. All other necropsy findings either occurred with similar frequencies in control and treated animals and are common findings in untreated animals and/or were considered related to the exsanguination procedure or were considered individual findings. The necropsy results are summarized in [Supplementary-material supplementary-material-1] in the Supplementary Material.

#### 3.4.5. Organ Weights

Testes and epididymide weights were statistically significantly lower compared to controls in the main 225 mg/kg group males as well as at the end of the recovery period in the 225 mg/kg recovery group males. In the main group the reduction was dose-related and also statistically significant for testes at 187 mg/kg and epididymides at 187 and 150 mg/kg. Epididymides weight relative to brain weight was statistically significantly lower in the 225, 187, and 150 mg/kg groups compared to controls and testes weight relative to brain weight was significantly lower compared to controls in the 225 mg/kg group. In the recovery group epididymide weight relative to body and brain weights were also statistically significantly lower compared to the corresponding controls.

Statistically significant reductions, often dose-related, in absolute brain, heart, spleen, and thymus weights in the main male groups compared to controls were without corresponding histopathology and were small in magnitude (remaining within or marginal to the historical control range) and were considered related to the depressed body weight development observed in the treated males, as were the statistically significant changes relative to body and/or brain weights in these organs as well as the adrenal glands. Following the recovery period, these weights were no longer significantly reduced, and in fact, were statistically significantly increased compared to the recovery control for spleen and thymus.

Statistically significant elevations in absolute liver weight occurred sporadically in the main male groups while increases in liver weight relative to body weight were dose-related and increased liver weight to brain weight ratio was statistically significant in the lower dose groups but was not significant in the 225 mg/kg males. Statistically significant increases were also observed for kidney weight relative to body weight (dose-related) and brain weight. A similar picture with respect to liver and kidney weights was observed in main group females. Absolute liver and kidney weights were statistically significantly elevated with respect to controls, but without a dose relationship. Significant elevations were also seen in liver and kidney weights relative to body weight and brain weight in all female groups with respect to controls and a dose relationship was apparent for both relative liver weights but was not apparent for the relative kidney weights. With the exception of kidney weight relative to body weight in males and liver weight relative to body and brain weights in females, the significant increases were recovered following 28 days without treatment. The changes remaining significant were lower in magnitude, suggesting partial recovery, and no correlating histopathological changes were observed; therefore, the liver and kidney weight findings were considered due to an adaptive response without toxicological relevance.

Absolute and relative to body and brain ovary weights were statistically significantly and dose-dependently increased in the top two or three main female dose groups. The changes were slight, remaining within or marginal to the historical control range, lacked correlating histopathology, and were fully recovered following 28 days without treatment. Therefore, they were not considered to be toxicologically relevant. Statistically significant decreases were observed with a dose-response in the main 225 mg/kg group females for absolute adrenal weight and for adrenal weight relative to body weight in the 225 and 187 mg/kg females. These changes also were within the historical control range, without correlating histopathology, and fully recovered in the high-dose females. A few additional sporadic statistically significant differences were observed in main group females for absolute brain, spleen, and thymus weights; spleen and thymus weights relative to body weight; and thymus weight relative to brain weight. These were minor changes within the historical control ranges, were without correlating histopathology, and, with the exception of thymus weights, were not significantly different from controls in the recovery group females. The organ weight results are summarized in Tables 8–10.

#### 3.4.6. Histopathology

**Figure 4:**
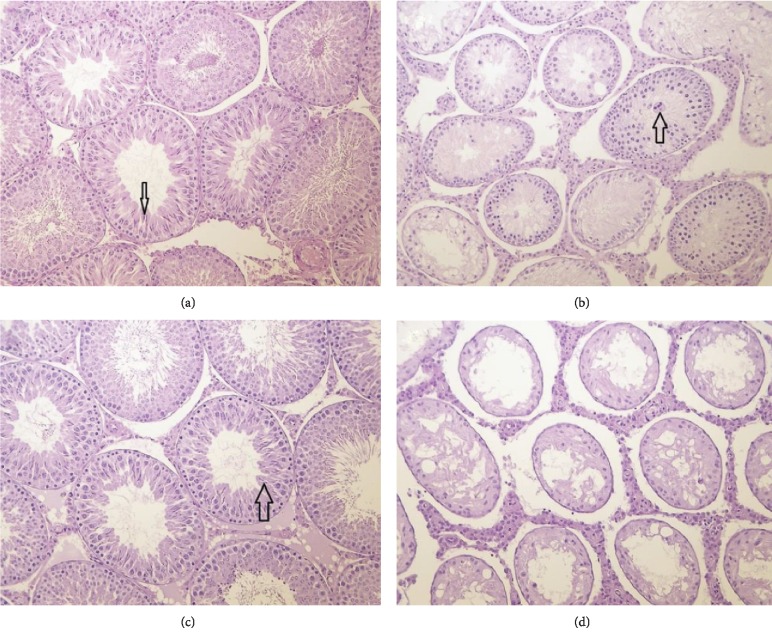
Testes sections of male rats in the 90-day study (H&E, 200X). (a) Normal active spermatogenesis and mature spermatozoa (arrow) in a male control animal at termination of treatment. (b) Decreased intensity of spermatogenesis, degeneration of germ cells, multinucleated giant cell (arrow), and lack of mature spermatozoa in a male animal at 225 mg/kg bw/day at termination of treatment. (c) Normal active spermatogenesis and mature spermatozoa (arrow) in a male animal at 225 mg/kg bw/day at the end of the recovery period. (d) Decreased intensity of spermatogenesis, degeneration of germ cells, and lack of mature spermatozoa in a male animal at 225 mg/kg bw/day at the end of the recovery period.

**Figure 5:**
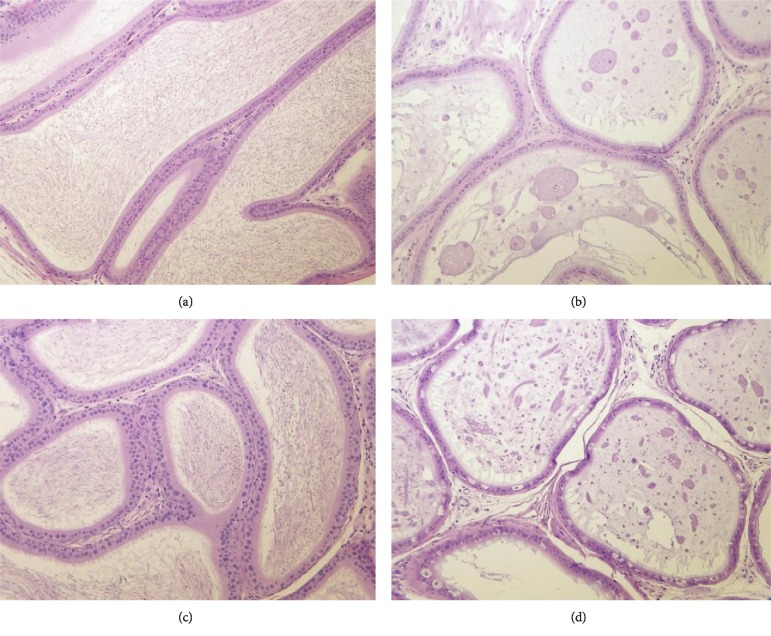
Epididymis sections of male rats in the 90-day study (H&E, 200X). (a) Normal storage of mature spermatozoa in a male control animal at termination of treatment. (b) Lack of mature spermatozoa in a male animal at 225 mg/kg bw/day at termination of treatment. (c) Normal storage of mature spermatozoa in a male control animal at 225 mg/kg bw/day at the end of the recovery period. (d) Lack of mature spermatozoa in a male control animal at 225 mg/kg bw/day at the end of the recovery period.

Histological examination revealed decreased intensity of spermatogenesis in the testes (see Figure 4(b)) and lack of mature spermatozoa in the epididymides (see Figure 5(b)) in the main group male animals at 225 mg/kg bw/day in correlation with macroscopic observations and organ weight changes of these organs. The alterations persisted in the recovery group males at 225 mg/kg bw/day at the end of the recovery period (Figures 4(d) and 5(d)). Because of the alterations observed at the high-dose, histopathological examination of the testes and epididymides was extended to the lower dose group animals. No alterations in the testes and epididymides were observed in the lower dose groups. In high-dose animals with severe (grade 4) lesions, mature spermatozoa were absent, and spermatids were detected in 90–100% of seminiferous tubuli; however, Sertoli-cells and spermatogonia were intact (Figure 4(b)). In moderate lesions (grade 3), 50–80% of tubuli contained spermatids and were absent of mature spermatozoa; in the unaffected tubuli, the developmental forms of spermatogenesis were intact. The intact spermatogonia and Sertoli-cells and the absence of inflammation or necrosis within the testes suggest a reversible character and the possibility of reactivation.

The number and cytomorphology of interstitial testicular cells were normal and the same as in control male animals. In the other animals at 225 mg/kg bw/day (4/10 at termination of the treatment and 2/5 at the end of the recovery period) and in all animals belonging to the lower dose groups (10/10/group) and control group (15/15) the various spermatogenic cells (the spermatogonia, the spermatocytes, the spermatids, and spermatozoa), representing different phases in the development and differentiation of the spermatozoa, and the interstitial cells were normal (Figures 4(a) and 4(c)). In all male animals, the histological picture of the prostate, seminal vesicle, and coagulating gland was normal as well. In all female animals of the treated and control groups, the histology of the ovaries, uterus, and vagina were normal.

Effects on spermatogenesis (and testicular atrophy) are well known with respect to the methylxanthines, and have been observed in rats, rabbits, and roosters and are generally thought to be reversible up on cessation of treatment [34]. Gans, observed these effects in caffeine- and theobromine-treated rats [32] while Weinberger et al. also observed them at high dietary doses in caffeine-, theobromine-, and theophylline-treated rats [35]. These effects, at least with respect to caffeine, have been largely attributed to suppression of follicle-stimulating hormone [34]. Testicular atrophy and inhibition of spermatogenesis have also been observed in rats treated with theacrine. Clewell et al. reported smaller than normal testes and epididymides, decreased intensity of spermatogenesis in the testes, and lack of mature spermatozoa in the epididymides in male animals dosed with theacrine at 300 mg/kg bw/day and above [30]. These histological findings are consistent with those observed with exposure to methylliberine in the current work. Of note, effects on male reproductive parameters, including fertility and semen quality, have not been convincingly associated with caffeine at typical levels of consumption in humans [36].

Other findings observed during the microscopic examination in the current work were of a nature common in experimental rats and occurred with similar frequencies in control and treated animals and/or were considered as individual findings or related to the exsanguination procedure. The histological findings are summarized in Table 11.

## 4. Conclusions

The test item did not induce frameshifts or base-pair substitution mutations in the genomes of the tester strains used under the applied conditions of the bacterial reverse mutation test nor did the test item induce chromosomal damage under the applied conditions of the in vitro chromosomal aberration or in vivo micronucleus tests. Based on the unequivocally negative results it is concluded that methylliberine is not mutagenic or clastogenic under the conditions of the conducted in vitro studies and does not cause in vivo genetic toxicity in the bone marrow of mice under the applied conditions.

In the 90-day study in rats, body weight development was depressed at 187 and 225 mg/kg bw/day and testicular atrophy and inhibition of spermatogenesis were observed at 225 mg/kg bw/day in male animals (testes weights were also reduced compared to controls at 187 mg/kg bw/day but were not grossly atrophic). These effects were not recovered in the high-dose animals 28 days after the final treatment. In female animals, no adverse effects were observed following repeated administration of the test item for 91 consecutive days. Thus, we conclude the NOAEL for Dynamine^®^ to be 150 and 225 mg/kg bw/day, respectively, in male and female Han:WIST rats.

## Figures and Tables

**Table 1:** Results of the initial mutation test.

Concentrations (*µ*g/plate)	*Salmonella typhimurium* tester strains																*Escherichia coli*			
	TA 98				TA 100				TA 1535				TA 1537				WP2uvrA			
	*−*S9		+S9		*−*S9		+S9		*−*S9		+S9		*−*S9		+S9		*−*S9		+S9	
Mean number of revertants per plate Mutation rate	Mean	MR	Mean	MR	Mean	MR	Mean	MR	Mean	MR	Mean	MR	Mean	MR	Mean	MR	Mean	MR	Mean	MR
Untreated Control	18.7	1.02	30.7	1.10	69.7	1.00	97.0	1.06	8.3	0.96	13.7	0.85	9.0	1.17	6.3	0.90	21.0	1.07	33.3	0.97
DMSO Control	18.3	1.00	28.0	1.00	70.0	1.00	91.3	1.00	8.7	1.00	16.0	1.00	7.7	1.00	7.0	1.00	19.7	1.00	34.3	1.00
*Ultrapure Water Control*	—	—	—	—	*79.3*	*1.00*	—	—	*8.7*	*1.00*	—	—	—	—	—	—	*22.3*	*1.00*	—	—
*Test item*																				
5000	22.3	1.22	17.3	0.62	27.7	0.40	21.3	0.23	8.7	1.00	7.7	0.48	5.0	0.65	8.7	1.24	20.7	1.05	20.0	0.58
1600	23.7	1.29	15.7	0.56	61.7	0.88	58.0	0.64	9.3	1.08	10.0	0.63	7.7	1.00	8.0	1.14	22.0	1.12	29.3	0.85
500	19.3	1.05	28.0	1.00	70.0	1.00	80.0	0.88	13.7	1.58	14.3	0.90	7.3	0.96	8.3	1.19	17.3	0.88	34.0	0.99
160	18.7	1.02	23.7	0.85	77.3	1.10	97.3	1.07	10.0	1.15	15.3	0.96	6.3	0.83	7.0	1.00	21.0	1.07	25.0	0.73
50	20.0	1.09	21.7	0.77	63.0	0.90	88.7	0.97	10.3	1.19	14.0	0.88	7.3	0.96	8.0	1.14	18.7	0.95	24.0	0.70
16	19.7	1.07	27.7	0.99	63.0	0.90	84.3	0.92	7.3	0.85	14.3	0.90	6.3	0.83	10.3	1.48	21.0	1.07	28.7	0.83
5	18.0	0.98	24.0	0.86	63.0	0.90	83.3	0.91	12.0	1.38	14.3	0.90	6.3	0.83	8.7	1.24	18.0	0.92	28.7	0.83
*Positive controls*																				
*NPD (4 μg/plate)*	*778.7*	*42.47*	—	—	—	—	—	—	—	—	—	—	—	—	—	—	—	—	—	—
*SAZ (2 μg/plate)*	—	—	—	—	*1546.7*	*19.50*	—	—	*1301.3*	*150.15*	—	—	—	—	—	—	—	—	—	—
*9AA (50 μg/plate)*	—	—	—	—	—	—	—	—	—	—	—	—	*396.0*	*51.65*	—	—	—	—	—	—
*MMS (2 μL/plate)*	—	—	—	—	—	—	—	—	—	—	—	—	—	—	—	—	*1600.0*	*71.64*	—	—
*2AA (2 μg/plate)*	—	—	*2213.3*	*79.05*	—	—	*2090.7*	*22.89*	—	—	*162.7*	*10.17*	—	—	*124.7*	*17.81*	—	—	—	—
*2AA (50 μg/plate)*	—	—	—	—	—	—	—	—	—	—	—	—	—	—	—	—	—	—	*164.7*	*4.80*

Abbreviations: MR, mutation rate; DMSO, dimethyl sulfoxide; NPD, 4-nitro-1,2-phenylenediamine; SAZ, sodium azide; 9AA, 9-aminoacridine; MMS, methyl methanesulfonate; 2AA, 2-aminoanthracene. Remarks: DMSO was applied as vehicle of the test item and the positive control substances: NPD, 9AA, and 2AA. Ultrapure water was applied as vehicle for the positive control substances: SAZ and MMS. The mutation rates of the test item, untreated control, NPD, 9AA, and 2AA are given referring to DMSO. The mutation rates of the SAZ and MMS positive controls are given referring to the ultrapure water.

**Table 2:** Results of the confirmatory mutation test.

	*Salmonella typhimurium* tester strains																*Escherichia coli*									
Concentrations (*µ*g/plate)	TA 98				TA 100				TA 1535				TA 1537				WP2uvrA			
	−S9		+S9		−S9		+S9		−S9		+S9		−S9		+S9		−S9		+S9	
Mean number of revertants per plate mutation rate	Mean	MR	Mean	MR	Mean	MR	Mean	MR	Mean	MR	Mean	MR	Mean	MR	Mean	MR	Mean	MR	Mean	MR
Untreated control	16.7	1.39	22.7	1.33	64.7	1.05	96.7	1.30	10.7	1.07	9.0	0.77	8.7	1.18	8.0	1.09	29.7	1.09	35.3	0.89
DMSO control	12.0	1.00	17.0	1.00	61.7	1.00	74.3	1.00	10.0	1.00	11.7	1.00	7.3	1.00	7.3	1.00	27.3	1.00	39.7	1.00
*Ultrapure water control*	—	—	—	—	*71.3*	*1.00*	—	—	*7.7*	*1.00*	—	—	—	—	—	—	*29.7*	*1.00*	—	—
*Test item*																																															
5000	16.0	1.33	17.0	1.00	26.3	0.43	24.7	0.33	8.7	0.87	7.7	0.66	10.7	1.45	8.7	1.18	31.7	1.16	34.0	0.86
1600	18.7	1.56	19.7	1.16	50.0	0.81	69.3	0.93	9.3	0.93	7.7	0.66	12.0	1.64	7.7	1.05	33.7	1.23	33.3	0.84
500	14.7	1.22	20.7	1.22	61.3	0.99	88.7	1.19	11.7	1.17	9.0	0.77	8.0	1.09	7.0	0.95	25.7	0.94	39.0	0.98
160	18.3	1.53	24.7	1.45	63.0	1.02	98.0	1.32	10.7	1.07	11.0	0.94	8.3	1.14	7.7	1.05	25.3	0.93	42.3	1.07
50	19.0	1.58	24.7	1.45	62.7	1.02	99.0	1.33	10.0	1.00	10.3	0.89	7.7	1.05	8.0	1.09	20.0	0.73	40.7	1.03
16	15.7	1.31	19.0	1.12	62.0	1.01	94.0	1.26	8.0	0.80	11.3	0.97	6.0	0.82	7.7	1.05	27.0	0.99	40.0	1.01
5	10.3	0.86	18.7	1.10	58.7	0.95	96.3	1.30	11.3	1.13	11.3	0.97	6.7	0.91	8.3	1.14	28.3	1.04	41.0	1.03
*Positive controls*																																															
*NPD (4 μg/plate)*	*490.0*	*40.83*	—	—	—	—	—	—	—	—	—	—	—	—	—	—	—	—	—	—
*SAZ (2 μg/plate)*	—	—	—	—	*1354.7*	*18.99*	—	—	*850.7*	*110.96*	—	—	—	—	—	—	—	—	—	—
*9AA (50 μg/plate)*	—	—	—	—	—	—	—	—	—	—	—	—	*333.0*	*45.41*	—	—	—	—	—	—
*MMS (2 μL/plate)*	—	—	—	—	—	—	—	—	—	—	—	—	—	—	—	—	*897.3*	*30.25*	—	—
*2AA (2 μg/plate)*	—	—	*1213.3*	*71.37*	—	—	*866.7*	*11.66*	—	—	*118.3*	*10.14*	—	—	*82.7*	*11.27*	—	—	—	—
*2AA (50 μg/plate)*	—	—	—	—	—	—	—	—	—	—	—	—	—	—	—	—	—	—	*164.3*	*4.14*

Abbreviations: MR, mutation rate; DMSO, dimethyl sulfoxide; NPD, 4-Nitro-1,2-phenylenediamine; SAZ, sodium azide; 9AA, 9-aminoacridine; MMS, methyl methanesulfonate; 2AA, 2-aminoanthracene. Remarks: DMSO was applied as vehicle of the test item and the positive control substances: NPD, 9AA, and 2AA. Ultrapure water was applied as vehicle for the positive control substances: SAZ and MMS. The mutation rates of the test item, untreated control, NPD, 9AA, and 2AA are given referring to DMSO. The mutation rates of the SAZ and MMS positive controls are given referring to the ultrapure water.

**Table 3:** Results of the in vitro mammalian chromosomal aberration test.

Experiment A																	
Groups		S9-mix	Treatment time	Sampling time	Cytotoxicity	Mean number of aberrant cells /150 cells		% of aberrant cells	Mean number of aberrations/150 cells		Mean aberrations						
											Chromosome			Chromatid			Others
						Gap+	Gap−	Gap-	Gap+	Gap−	Gap	Del	Exchange	Gap	Del	Exchange	
*Test item*																	
250 µg/mL		−	3 h	20 h	5	7	4	2.333	7	4	0	0	0	4	4	0	—
500 µg/mL		−	3 h	20 h	16	7	4	2.667	7	4	2	1	1	1	2	1	—
1000 µg/mL		−	3 h	20 h	27	9	5	3.000	9	5	1	1	1	4	3	0	—
2000 µg/mL		−	3 h	20 h	32	8	3	2.000	8	3	3	0	1	3	2	0	—
Vehicle control		−	3 h	20 h	0	8	4	2.667	8	4	1	0	2	3	2	0	—
Positive control		−	3 h	20 h	50	39^∗∗^	33^∗∗^	22.000	57^∗∗^	38^∗∗^	7	8	6	12	13	12	—
*Test item*																	
250 µg/mL		+	3 h	20 h	6	8	5	3.000	9	6	2	1	2	2	1	3	—
500 µg/mL		+	3 h	20 h	17	10	4	2.667	10	4	3	1	1	4	2	1	—
1000 µg/mL		+	3 h	20 h	20	9	5	3.000	9	5	1	1	1	4	1	2	—
2000 µg/mL		+	3 h	20 h	32	8	4	2.667	8	4	1	1	1	4	3	0	—
Vehicle control		+	3 h	20 h	0	8	6	3.667	9	6	1	1	3	3	2	1	—
Positive control		+	3 h	20 h	52	44^∗∗^	38^∗∗^	25.333	88^∗∗^	59^∗∗^	10	8	7	19	11	34	—
Experiment B																	
*Test item*																	
125 µg/mL	−		20 h	20 h	6	7	4	2.333	8	4	1	1	1	3	2	1	—
250 µg/mL	−		20 h	20 h	20	9	4	2.667	9	4	1	1	1	4	2	1	—
500 µg/mL	−		20 h	20 h	56	8	3	2.000	8	3	2	1	1	3	1	0	—
Vehicle control	−		20 h	20 h	0	6	3	2.000	6	3	1	0	1	2	2	0	—
Positive control	−		20 h	20 h	52	41^∗∗^	34^∗∗^	22.667	67^∗∗^	46^∗∗^	8	7	7	13	13	20	—
*Test item*																	
125 µg/mL	−		20 h	28 h	6	6	3	2.000	6	3	1	0	2	2	2	0	—
250 µg/mL	−		20 h	28 h	22	7	3	2.667	8	3	2	1	0	3	2	1	—
500 µg/mL	−		20 h	28 h	56	8	4	2.667	9	4	1	0	2	4	3	0	—
Vehicle control	−		20 h	28 h	0	7	4	2.333	7	4	1	0	2	3	2	1	—
Positive control	−		20 h	28 h	49	46^∗∗^	41^∗∗^	27.000	73^∗∗^	48^∗∗^	7	7	7	18	16	20	—
*Test item*																	
250 µg/mL	+		3 h	28 h	4	7	3	2.000	7	3	0	1	1	4	2	0	—
500 µg/mL	+		3 h	28 h	23	8	4	2.333	8	4	2	0	1	3	2	1	—
1000 µg/mL	+		3 h	28 h	26	8	3	2.000	8	3	2	0	1	4	2	1	—
2000 µg/mL	+		3 h	28 h	33	9	4	2.333	10	4	2	1	2	4	1	0	—
Vehicle control	+		3 h	28 h	0	7	3	2.000	7	3	1	0	2	4	2	0	—
Positive control	+		3 h	28 h	53	45^∗∗^	42^∗∗^	27.667	89^∗∗^	62^∗∗^	8	9	9	20	17	28	—

Abbreviations: −, without S9-mix; +, with S9=mix; del deletion; gap−, excluding gaps; gap+, including gaps. Vehicle control, DME medium; positive control with S9-mix, cyclophasphamide (5.0 *µ*g/mL); positive control without S9-mix (experiment A), EMS (1.0 *µ*g/mL); positive control without S9-mix (experiment B), EMS (0,4 *µ*g/mL). Cytotoxicity=100-RICC (relative increase in cell counts). ^∗∗^p < 0.01.

**Table 4:** Results of the in vivo mammalian micronucleus test.

Groups (*n* = 5^^†^^)	Sampling time (hours following final treatment)	Total number of PCEs analysed	MPCE (per 4000 PCE)		PCE/PCE + NCE	
			Mean	±SD	Mean	±SD
Historical negative control	24	260000	5.05	1.00	—	—
Concurrent negative control	24	20000	6.20	1.30	0.53	0.02
175 mg/kg bw	24	20000	5.80	1.64	0.52	0.01
350 mg/kg bw	24	20000	6.20	1.30	0.50	0.02
700 mg/kg bw	24	20000	6.80^∗^	1.30	0.49	0.01
Positive control (60 mg/kg bw)	24	20000	141.00^∗∗^	5.96	0.39	0.05

^^†^^Historical negative control (*n* = 65). ∗*p* < 0.05 to the historical negative control (value was inside in the 95% control limits (2.87–7.23) of the historical control data). ^∗∗^*p* < 0.01 to the concurrent and historical negative control.

**Table 5:** Body weight gain in the 90-day study.

Group (mg/kg bw/day)		Body weight gain (g) between days																						
		Treatment period																		Recovery Period				
		0–4	4–7	7–11	11–14	14–18	18–21	21–25	25–28	28–35	35–42	42–49	49–56	56–63	63–70	70–77	77–84	84–89	0–89	90–97	97–104	104–111	111–117	90–117
*Male*																								
0 (Control)	Mean	27.3	17.7	24.1	14.5	19.1	10.1	15.2	10.1	20.6	15.4	14.7	14.1	9.6	6.9	10.9	7.3	2.3	239.9	9.8	4.0	3.4	10.2	27.4
(*n* = 15^†^)	SD	2.9	3.5	3.0	2.6	3.3	2.1	3.2	3.1	3.8	3.4	5.3	3.9	2.9	2.9	3.7	4.0	3.8	24.9	3.3	1.6	3.9	1.1	6.3
75	Mean	25.0	15.5	18.9	13.1	18.9	6.3	17.0	8.3	22.7	16.0	15.3	15.3	11.7	8.1	11.0	9.4	1.5	234.0					
(*n* = 10)	SD	3.7	4.0	5.2	2.0	5.6	3.4	3.6	2.7	4.9	5.3	5.4	4.1	6.2	4.3	4.0	3.3	3.1	36.3					
	SS			^∗∗^			^∗∗^																	
112	Mean	23.9	15.8	22.4	12.6	21.0	9.7	16.8	8.7	21.8	16.3	14.1	14.6	9.2	6.8	10.9	9.7	−0.3	234.0					
(*n* = 10)	SD	4.7	3.0	3.7	2.6	3.9	2.3	3.9	2.0	3.5	7.0	3.4	7.1	3.7	4.6	4.7	4.6	4.4	20.4					
	SS	^∗^																						
150	Mean	20.7	15.5	18.3	14.4	16.8	7.1	16.6	3.8	21.2	13.7	11.5	12.5	10.0	4.6	10.3	6.7	3.2	206.9					
(*n* = 10)	SD	5.2	3.9	2.9	1.6	4.7	4.5	2.9	3.5	4.5	3.7	5.1	2.6	3.7	3.3	5.0	4.7	3.3	24.9					
	SS	^∗∗^		^∗∗^			^∗^		^∗∗^										^∗∗^					
187	Mean	19.5	14.0	15.5	10.2	16.4	7.1	17.0	5.5	20.2	13.3	6.5	13.3	9.3	6.0	7.1	6.1	3.8	190.8					
(*n* = 10)	SD	4.4	3.5	4.3	4.1	4.8	5.5	3.5	3.7	8.1	3.1	2.9	4.9	4.2	5.3	3.2	3.5	4.5	27.9					
	SS	^∗∗^	^∗^	^∗∗^	^∗∗^				^∗∗^			^∗∗^							^∗∗^					
225	Mean	17.3	11.9	13.0	8.7	11.9	6.2	13.9	4.7	16.3	7.9	7.8	9.7	5.2	5.7	8.0	6.8	1.7	156.5	15.2	23.4	15.4	9.6	63.6
(*n* = 15^†^)	SD	2.8	4.0	3.8	3.5	3.5	3.3	2.9	2.7	5.3	3.3	4.1	4.4	4.8	4.3	4.5	3.9	3.6	25.0	7.7	7.5	6.4	3.9	23.1
	SS	^∗∗^	^∗∗^	^∗∗^	^∗∗^	^∗∗^	^∗∗^		^∗∗^	^∗^	^∗∗^	^∗∗^	^∗^	^∗^					^∗∗^		^∗∗^	^∗∗^		^∗^
Test for significance		DN	DN	DN	DN	DN	*U*	NS	DN	DN	*U*	DN	DN	DN	NS	NS	NS	NS	DN	NS	*T*	*T*	NS	*T*
*Female*																								
0 (Control)	Mean	13.2	6.6	12.4	5.7	10.1	5.5	6.3	4.9	7.5	8.4	5.6	3.1	6.5	3.4	0.9	4.7	3.6	108.3	−0.6	3.2	−1.0	1.8	3.4
(*n* = 15^†^)	SD	2.4	2.6	2.9	4.5	2.3	4.9	2.3	2.9	2.7	4.7	3.9	3.1	2.5	4.2	3.0	3.8	3.7	14.6	4.5	4.7	2.7	3.3	6.5
75	Mean	13.1	6.5	12.3	5.8	9.9	3.3	6.6	3.5	12.0	7.0	6.4	5.7	7.7	3.4	1.7	3.2	4.4	112.5					
(*n* = 10)	SD	4.1	3.7	2.8	3.2	2.5	2.7	3.3	3.0	4.0	3.8	4.4	5.1	1.7	3.9	3.9	4.7	3.6	8.2					
	SS									^∗∗^														
112	Mean	13.1	8.1	11.1	5.8	9.4	3.7	6.7	3.6	10.7	8.0	7.3	6.0	5.6	4.8	−0.1	3.0	6.0	112.8					
(*n* = 10)	SD	3.8	3.0	3.6	2.0	2.7	3.7	2.5	3.6	4.1	3.0	4.9	3.7	3.9	3.9	5.1	2.1	4.1	12.6					
	SS									^∗^			^∗^											
150	Mean	11.6	9.2	11.6	6.3	9.6	2.9	8.1	3.0	12.5	7.1	6.7	7.9	3.7	3.4	4.3	4.4	1.8	114.1					
(*n* = 10)	SD	2.2	2.9	2.0	2.4	1.8	4.0	2.2	3.6	3.8	3.5	2.8	2.1	2.2	3.0	3.7	3.6	2.6	14.8					
	SS									^∗∗^			^∗∗^	^∗^										
187	Mean	13.0	8.4	12.8	6.9	9.8	2.5	9.6	2.3	10.3	8.3	5.2	6.5	6.2	3.6	5.3	4.1	3.7	118.5					
(*n* = 10)	SD	2.1	1.8	5.3	2.8	3.2	3.2	2.4	2.3	2.5	1.6	3.3	2.4	2.3	4.2	4.0	2.8	2.6	17.4					
	SS							^∗^		^∗^			^∗^			^∗^								
225	Mean	10.1	8.3	11.0	4.4	8.9	4.0	8.7	3.0	10.9	5.9	4.8	8.1	4.4	4.3	2.5	3.1	2.1	104.6	−5.4	4.6	0.4	0.6	0.2
(*n* = 15^†^)	SD	2.1	2.7	3.5	3.6	3.3	2.1	3.7	4.6	6.3	4.2	3.0	2.7	2.3	2.7	4.2	3.2	4.3	14.6	2.6	5.0	3.2	2.1	5.8
	SS	^∗∗^						^∗^					^∗∗^	^∗^										
Test for significance		DN	NS	NS	NS	NS	NS	DN	NS	*U*	NS	NS	DN	DN	NS	DN	NS	NS	NS	NS	NS	NS	NS	NS

Abbreviations: DN, Duncan's multiple range test; NS, not significant; SD, standard deviation; SS, statistically significant compared to control; *TT*-test versus control; *U*, Mann-Whitney *U*-test versus control.

^†^Recovery period (*n* = 5). ^∗^*p* < 0.05, ^∗∗^*p* < 0.01.

**Table 6:** Results of the hematological evaluation in the 90-day study.

Group (mg/kg bw/day)		WBC	NEU	LYM	MONO	EOS	BASO	RBC	HGB	HCT	MCV	MCH	MCHC	PLT	RET	PT	APTT
		[×10^9^/L]	[%]	[%]	[%]	[%]	[%]	[×10^12^/L]	[g/L]	[L/L]	[fL]	[pg]	[g/L]	[×10^9^/L]	[%]	[sec]	[sec]
*Main study groups*																	
*Male*																	
0 (Control)	Mean	7.01	17.76	78.20	2.14	1.31	0.08	9.35	160.0	0.49	51.94	17.09	329.4	907.8	1.67	10.44	13.54
(*n* = 10)	SD	1.51	7.28	7.59	0.66	0.36	0.06	0.41	6.9	0.02	1.01	0.40	3.5	78.7	0.29	0.21	1.13
75	Mean	7.67	20.32	74.96	2.81	1.10	0.08	9.02	162.3	0.49	54.05	18.02	333.5	849.4	2.23	10.46	14.39
	SD	1.32	3.01	3.67	0.66	0.47	0.06	0.46	4.1	0.02	1.18	0.56	7.5	104.0	0.29	0.25	1.05
	SS		^∗^								^∗∗^	^∗∗^			^∗∗^		
112	Mean	8.58	25.13	69.95	2.86	1.30	0.08	9.00	165.00	0.50	55.27	18.36	332.30	896.20	2.74	10.10	13.91
(*n* = 10)	SD	2.19	15.45	15.92	0.69	0.52	0.04	0.30	3.23	0.01	1.67	0.61	3.83	75.65	0.52	0.24	1.52
	SS		^∗^	^∗^							^∗∗^	^∗∗^			^∗∗^	^∗∗^	
150	Mean	9.22	29.51	65.60	2.81	1.42	0.09	9.09	168.50	0.51	55.79	18.55	332.50	882.80	2.73	10.12	13.62
(*n* = 10)	SD	2.83	12.06	14.00	0.74	1.81	0.07	0.45	6.10	0.02	2.38	0.82	4.14	121.32	0.37	0.16	1.63
	SS		^∗∗^	^∗∗^		^∗^			^∗∗^	^∗^	^∗∗^	^∗∗^			^∗∗^	^∗∗^	
187	Mean	9.08	26.57	69.04	3.00	0.77	0.11	9.01	168.50	0.52	57.27	18.71	327.00	896.70	3.15	10.27	15.17
(*n* = 10)	SD	1.99	3.61	3.81	0.50	0.20	0.03	0.30	4.33	0.02	1.31	0.43	4.45	115.59	0.30	0.26	0.87
	SS		^∗∗^	^∗∗^		^∗∗^			^∗∗^	^∗∗^	^∗∗^	^∗∗^			^∗∗^		^∗^
225	Mean	7.89	31.96	63.34	3.04	0.94	0.09	9.07	170.10	0.52	57.35	18.81	327.90	933.90	3.27	10.23	15.17
(*n* = 10)	SD	2.39	9.43	10.88	1.20	0.42	0.03	0.53	6.69	0.02	2.36	0.83	5.95	84.10	0.32	0.21	1.46
	SS		^∗∗^	^∗∗^		^∗^			^∗∗^	^∗∗^	^∗∗^	^∗∗^			^∗∗^		^∗^
Test for significance		NS	*U*	*U*	NS	*U*	NS	NS	DN	DN	DN	DN	NS	NS	DN	DN	DN
Historical control Range		4.3–10.6	10-3–35.7	60.1–86.5	1.2–6.7	0.7–2.8	0.0–0.2	8.1–9.9	145.0–183.0	0.4–0.5	49.9–60.2	16.3–20.7	315.0–361.0	339.0–1062.0	1.2–2.3	10.0–11.2	10.2–15.6

*Female*																	
0 (Control)	Mean	4.91	21.03	74.11	2.52	1.89	0.08	8.41	154.6	0.46	55.16	18.39	333.6	886.7	1.73	9.91	13.35
(*n* = 10)	SD	1.72	13.74	14.85	0.67	0.96	0.06	0.27	4.6	0.01	1.14	0.44	5.5	174.7	0.36	0.21	1.28
75	Mean	5.98	17.53	78.14	2.64	1.14	0.07	8.12	150.8	0.46	56.85	18.59	327.2	905.8	2.55	9.93	15.15
(*n* = 10)	SD	1.65	4.21	3.88	0.76	0.59	0.05	0.35	4.9	0.02	1.32	0.74	8.0	104.7	0.40	0.37	1.17
	SS					^∗^					^∗^		^∗^		^∗∗^		^∗∗^
112	Mean	6.27	18.41	77.83	2.37	0.91	0.07	8.22	152.1	0.46	55.47	18.52	333.6	947.7	2.84	9.88	15.19
(*n* = 10)	SD	1.80	5.20	5.12	0.52	0.16	0.05	0.41	8.7	0.02	0.84	0.58	8.0	97.7	0.38	0.09	1.11
	SS					^∗∗^									^∗∗^		^∗∗^
150	Mean	7.24	22.44	73.93	2.50	0.61	0.06	8.14	153.4	0.46	57.08	18.86	330.4	927.5	3.04	9.85	14.37
(*n* = 10)	SD	1.18	4.07	3.92	0.31	0.10	0.05	0.31	3.7	0.01	1.13	0.50	4.8	115.8	0.42	0.20	1.28
	SS	^∗∗^				^∗∗^					^∗∗^				^∗∗^		
187	Mean	7.39	22.00	74.21	2.58	0.60	0.10	8.19	151.4	0.46	56.60	18.52	327.2	967.4	3.27	9.80	13.67
(*n* = 9^†^)	SD	1.48	7.11	7.31	0.84	0.14	0.00	0.46	9.2	0.02	1.88	0.59	5.7	100.8	0.39	0.22	1.46
	SS	^∗∗^				^∗∗^					^∗^		^∗^		^∗∗^		
225	Mean	7.53	25.68	70.03	2.96	0.74	0.06	8.21	153.4	0.46	56.37	18.72	332.0	1013.4	3.15	9.85	14.14
(*n* = 10)	SD	1.49	12.34	13.37	0.96	0.40	0.05	0.36	5.7	0.01	1.49	0.68	5.3	94.2	0.53	0.13	0.91
	SS	^∗∗^	^∗^	^∗^		^∗∗^									^∗∗^		
Test for significance		DN	*U*	*U*	NS	*U*	NS	NS	NS	NS	DN	NS	DN	NS	DN	NS	DN
Historical control range		1.5–9.2	7.6–50.2	43.7–88.7	1.0–5.0	0.8–3.7	0.0–0.2	7.4–10.1	141.0–175.0	0.4–0.5	50.1–57.7	17.2–20.1	319.0–365.0	450.0–1127.0	1.2–3.6	9.5–10.5	11.1–16.1
*Recovery groups*																	
*Male*																	
0 (Control)	Mean	7.30	18.02	76.32	3.06	1.90	0.12	8.88	156.40	0.48	53.32	17.60	330.2	786.4	1.63	10.68	12.22
(*n* = 5)	SD	2.26	4.50	3.98	1.83	1.08	0.04	0.39	7.92	0.02	0.83	0.23	8.0	155.9	0.21	0.20	1.19
225	Mean	7.88	19.98	75.06	3.04	1.28	0.12	8.56	159.20	0.50	57.80	18.62	321.8	758.6	2.47	10.70	12.96
(*n* = 5)	SD	0.63	5.23	5.70	0.88	0.33	0.04	0.27	3.70	0.02	1.24	0.40	2.2	119.5	0.30	0.22	0.54
	SS										^∗∗^	^∗∗^			^∗∗^		
Test for significance		NS	NS	NS	NS	NS	NS	NS	NS	NS	*T*	*T*	NS	NS	*T*	NS	NS
*Female*																	
0 (Control)	Mean	4.20	25.48	68.94	2.52	2.62	0.08	8.68	155.20	0.48	54.82	17.88	326.0	740.4	2.22	10.10	13.14
(*n* = 5)	SD	1.41	5.24	4.70	0.47	2.06	0.08	0.34	4.92	0.02	0.95	0.41	7.9	169.5	0.20	0.33	1.06
225	Mean	5.18	20.80	75.42	2.08	1.16	0.08	8.69	156.60	0.48	54.92	18.04	328.4	863.0	1.78	10.12	14.32
(*n* = 5)	SD	1.06	5.56	5.42	0.46	0.18	0.04	0.45	4.98	0.02	1.40	0.51	4.3	145.6	0.38	0.13	0.57
Test for significance		NS	NS	NS	NS	NS	NS	NS	NS	NS	NS	NS	NS	NS	NS	NS	NS

Abbreviations: APTT, activated partial thromboplastin time; BASO, basophil granulocytes; DN, Duncan's multiple range test; EOS, eosinophil granulocytes; HCT, hematocrit; HGB, hemoglobin; LYM, lymphocyte; MCV, mean corpuscular volume; MCH, mean corpuscular hemoglogin; MCHC, mean corpuscular hemoglobin concentration; MONO, monocyte; NEU, neutrophil granulocytes; NS, not significant; PLT, platelet count; PT prothrombin time; RBC, red blood cell (erythrocyte); RET, reticulocyte; SD, standard deviation; SS, statistically significant compared to control; *TT*-test versus control; *U*, Mann-Whitney *U*-test versus control; WBC, white blood cell. ^†^Main group females at 187 mg/kg bw/day (*n* = 9; one animal died at collection due to over anesthesia). ^∗^*p* < 0.05, ^∗∗^*p* < 0.01.

**Table 7:** Results of the clinical chemistry evaluation in the 90-day study.

Group (mg/kg bw/day)		ALT	AST	ALP	TBIL	CREA	UREA	GLUC	CHOL	Pi	Ca^++^	Na^+^	K^+^	Cl^−^	ALB	TPROT	A/G
		[U/L]	[U/L]	[U/L]	[*µ*mol/L]	[*µ*mol/L]	[mmol/L]	[mmol/L]	[mmol/L]	[mmol/L]	[mmol/L]	[mmol/L]	[mmol/L]	[mmol/L]	[g/L]	[g/L]	
*Main study groups* (*n* = 10^†^)																	
*Male*																	
0 (Control)	Mean	40.9	95.3	87.7	1.07	29.7	7.52	6.09	2.39	1.91	2.60	144.53	4.37	100.32	44.20	63.99	2.25
(*n* = 10)	SD	5.5	15.1	12.8	0.32	3.2	0.82	0.43	0.23	0.16	0.07	1.30	0.21	0.81	1.06	1.79	0.12
75	Mean	50.5	78.9	74.6	1.12	25.7	7.90	5.77	2.87	2.05	2.63	143.78	4.61	98.77	45.02	64.86	2.28
(*n* = 10)	SD	15.8	8.3	16.7	0.29	3.4	0.93	0.55	0.29	0.17	0.06	0.58	0.33	1.41	1.38	2.86	0.19
	SS		^∗∗^	^∗^		^∗^			^∗∗^				^∗^	^∗∗^			
112	Mean	40.9	87.7	69.3	1.11	29.8	7.94	5.48	3.27	2.14	2.66	143.80	4.58	97.83	45.05	65.78	2.20
(*n* = 10)	SD	3.8	15.2	10.0	0.31	4.6	1.00	0.65	0.35	0.19	0.09	2.29	0.23	2.74	1.36	2.18	0.26
	SS			^∗∗^					^∗∗^	^∗^				^∗^			
150	Mean	47.5	89.9	68.5	0.95	30.0	7.24	5.68	3.67	2.27	2.71	144.10	4.35	97.70	44.81	64.05	2.36
(*n* = 10)	SD	9.3	7.4	13.1	0.21	3.3	0.99	0.82	0.39	0.06	0.09	0.84	0.20	0.96	2.19	3.12	0.27
	SS			^∗∗^					^∗∗^	^∗∗^	^∗∗^			^∗∗^			
187	Mean	52.9	83.9	63.9	1.22	27.6	8.06	6.01	3.63	1.99	2.62	142.86	4.32	97.28	45.89	64.42	2.47
(*n* = 10)	SD	9.5	9.5	17.5	0.47	2.7	1.53	0.90	0.63	0.29	0.09	1.42	0.12	1.94	0.83	1.62	0.21
	SS	^∗∗^	^∗^	^∗∗^					^∗∗^			^∗^		^∗∗^	^∗^		^∗^
225	Mean	58.3	85.3	57.9	1.06	29.6	7.00	5.29	4.12	2.12	2.63	141.53	3.96	95.90	45.97	65.33	2.38
(*n* = 10)	SD	10.0	10.0	15.6	0.23	3.1	0.58	0.64	0.38	0.15	0.09	1.69	0.22	1.55	1.63	2.91	0.22
	SS	^∗∗^		^∗∗^				^∗^	^∗∗^	^∗∗^		^∗∗^	^∗∗^	^∗∗^	^∗^		
Test for significance		DN	DN	DN	NS	DN	NS	DN	DN	*U*	DN	*U*	DN	*U*	DN	NS	DN
Historical control range		26.0–70.0	65.0–131.0	62.0–209.0	0.4–2.5	20.0–35.0	3.3–8.9	4.7–9.2	1.4–3.1	1.5–2.3	2.4–2.9	141.2–148.4	4.1–5.2	96.8–103.2	40.1–47.3	59.9–70.1	1.5–2.6
*Female*																	
0 (Control)	Mean	48.7	99.7	41.5	1.84	29.0	6.51	5.29	2.20	1.83	2.67	143.59	4.24	101.08	49.61	66.44	3.00
(*n* = 10)	SD	12.5	21.0	9.5	0.81	2.7	0.68	0.63	0.25	0.12	0.09	1.07	0.21	1.60	3.17	4.73	0.44
75	Mean	52.0	101.6	50.9	0.92	27.4	7.04	6.31	3.03	1.93	2.67	142.25	4.41	99.30	48.07	66.40	2.63
(*n* = 10)	SD	9.0	20.2	29.6	0.34	3.1	0.74	0.97	0.93	0.35	0.09	2.08	0.39	1.75	3.82	5.40	0.27
	SS				^∗∗^			^∗∗^	^∗^			^∗^		^∗^			
112	Mean	46.0	84.6	37.8	1.15	26.1	6.79	6.15	3.53	1.94	2.68	141.86	4.16	97.77	48.40	67.56	2.53
(*n* = 10)	SD	10.8	22.8	14.0	0.40	3.1	0.94	1.08	0.59	0.36	0.10	1.21	0.31	1.28	2.99	4.82	0.21
	SS				^∗∗^			^∗^	^∗∗^			^∗∗^		^∗∗^			^∗^
150	Mean	50.0	84.2	53.5	1.03	24.4	7.09	6.70	3.02	2.09	2.76	141.47	4.41	96.13	48.75	66.46	2.75
(*n* = 10)	SD	12.2	16.7	18.9	0.44	3.6	0.72	0.59	0.65	0.17	0.09	1.17	0.30	1.57	2.34	3.56	0.23
	SS				^∗∗^	^∗∗^		^∗∗^	^∗∗^	^∗∗^	^∗^	^∗∗^		^∗∗^			
187	Mean	56.3	80.3	39.6	1.23	25.9	7.12	6.39	3.55	2.33	2.76	140.38	4.48	95.94	46.89	64.21	2.77
(*n* = 9^^†^^)	SD	7.3	6.2	7.8	0.40	3.1	1.59	0.62	1.02	0.19	0.10	1.03	0.19	2.09	2.89	4.49	0.38
	SS		^∗^		^∗^	^∗^		^∗∗^	^∗∗^	^∗∗^		^∗∗^		^∗∗^			
225	Mean	50.2	76.6	36.4	1.24	25.0	6.73	6.18	3.45	2.29	2.77	140.89	4.42	95.69	48.25	65.56	2.79
(*n* = 10)	SD	13.4	8.9	10.3	0.37	3.0	1.25	0.51	0.83	0.14	0.09	0.75	0.26	1.58	1.65	2.37	0.20
	SS		^∗∗^		^∗∗^	^∗^		^∗^	^∗^	^∗∗^	^∗^	^∗∗^		^∗∗^			
Test for significance		NS	*U*	NS	DN	DN	NS	DN	*U*	*U*	DN	DN	NS	DN	NS	NS	DN
Historical control range		28.0–133.0	66.0–249.0	22.0–162.0	0.5–3.6	24.0–40.0	3.8–9.5	4.0–7.3	1.1–2.8	0.8–2.1	2.4–2.9	140.9–146.5	3.1–4.6	97.6–105.0	43.8–57.6	56.5–78.9	1.7–3.7
*Recovery groups*																	
*Male*																	
0 (Control)	Mean	46.80	96.60	83.60	1.66	28.60	7.60	6.09	2.20	2.11	2.67	144.66	4.63	100.30	42.96	62.88	2.18
(*n* = 5)	SD	6.38	22.70	17.29	0.40	3.36	1.30	1.00	0.15	0.09	0.04	1.41	0.21	1.36	0.97	2.00	0.28
225	Mean	40.20	72.60	71.60	1.46	27.60	6.64	5.36	2.45	2.23	2.68	143.64	4.68	99.22	43.16	62.54	2.24
(*n* = 5)	SD	2.39	8.26	11.01	0.29	1.14	0.31	0.25	0.36	0.11	0.02	0.34	0.11	0.57	1.37	0.90	0.23
Test for significance		NS	NS	NS	NS	NS	NS	NS	NS	NS	NS	NS	NS	NS	NS	NS	NS
*Female*																	
0 (Control)	Mean	48.80	95.20	37.80	1.84	36.80	6.76	5.64	1.94	1.39	2.59	141.36	4.04	99.44	48.56	66.56	2.72
(*n* = 5)	SD	9.63	13.08	13.77	0.43	5.76	1.09	0.37	0.38	0.21	0.05	1.05	0.29	0.76	2.25	3.77	0.28
225	Mean	37.80	77.80	36.40	1.88	30.40	6.72	5.43	2.48	1.39	2.65	142.02	4.23	99.58	48.86	67.90	2.64
(*n* = 5)	SD	4.60	24.24	22.86	0.84	2.97	1.05	0.82	0.64	0.46	0.08	0.98	0.19	1.91	3.16	4.10	0.45
Test for significance		NS	NS	NS	NS	NS	NS	NS	NS	NS	NS	NS	NS	NS	NS	NS	NS

Abbreviations: A/G, albumin to globulin ratio; ALB, albumin; ALP, alkaline phosphatase; ALT, alanine aminotransferase; AST, aspartate aminotransferase; BAC, bile acids; Ca++, calcium; CHOL, cholesterol; Cl−, chloride; CREA, creatinine; DN, Duncan's multiple range test; GLUC, glucose; K+, potassium; Na+, sodium; NS, Not Significant; Pi, inorganic phosphorous; SD, standard deviation; SS, statistically significant compared to control; TBIL, total bilirubin; TPROT, total protein; *U*, Mann-Whitney *U*-test versus control. ^†^Main group females at 187 mg/kg bw/day (*n* = 9; one animal died at collection due to over anesthesia). ^∗^*p* < 0.05, ^∗∗^*p* < 0.01.

**Table 8:** Organ weights in the 90-day study.

Group (mg/kg bw/day)		Organ weight (g)									
		Body weight	Brain	Liver	Kidneys	Heart	Spleen	Thymus	Testes	Epididymides	Adrenal glands
*Main study groups *(*n* = 10)											
*Male*											
0 (Control)	Mean	408.8	2.17	9.76	2.27	1.06	0.66	0.40	3.57	1.49	0.068
(*n* = 10)	SD	25.21	0.07	0.81	0.15	0.07	0.10	0.06	0.21	0.08	0.005
75	Mean	411.1	2.13	10.79	2.51	1.04	0.71	0.35	3.69	1.45	0.072
(*n* = 10)	SD	29.76	0.09	0.77	0.37	0.10	0.08	0.04	0.27	0.21	0.010
	SS			^∗^				^∗^			
112	Mean	407.6	2.14	11.01	2.56	1.03	0.68	0.30	3.53	1.37	0.067
(*n* = 10)	SD	17.09	0.08	0.70	0.27	0.10	0.08	0.06	0.33	0.21	0.010
	SS			^∗∗^				^∗∗^			
150	Mean	378.5	2.07	10.43	2.50	0.96	0.59	0.26	3.26	1.23	0.068
(*n* = 10)	SD	27.32	0.08	1.00	0.24	0.06	0.09	0.04	0.38	0.15	0.014
	SS	^∗^	^∗^			^∗^		^∗∗^		^∗∗^	
187	Mean	363.0	2.04	10.48	2.52	0.93	0.60	0.26	3.25	1.19	0.068
(*n* = 10)	SD	26.92	0.11	0.95	0.31	0.10	0.08	0.06	0.24	0.16	0.010
	SS	^∗∗^	^∗∗^			^∗∗^		^∗∗^	^∗∗^	^∗∗^	
225	Mean	317.5	2.03	9.67	2.41	0.85	0.54	0.22	2.00	1.06	0.067
(*n* = 10)	SD	17.13	0.10	0.97	0.20	0.08	0.08	0.04	0.93	0.22	0.014
	SS	^∗∗^	^∗∗^			^∗∗^	^∗∗^	^∗∗^	^∗∗^	^∗∗^	
Test for significance		DN	DN	DN	NS	DN	DN	DN	*U*	DN	NS
Historical control range		363.0–548.0	2.00–2.35	8.20–14.60	1.95–3.19	0.93–1.37	0.51–0.93	0.25–0.59	2.99–4.43	1.20–1.19	0.041–0.091

*Female*									*Ovaries*	*Uterus*	
0 (Control)	Mean	235.9	1.93	5.95	1.52	0.70	0.45	0.33	0.61	0.091	0.081
(*n* = 10)	SD	10.95	0.08	0.54	0.09	0.04	0.05	0.05	0.12	0.016	0.012
75	Mean	243.1	2.00	7.42	1.70	0.73	0.52	0.29	0.71	0.102	0.080
(*n* = 10)	SD	8.70	0.09	0.67	0.07	0.05	0.05	0.06	0.14	0.019	0.008
	SS			^∗∗^	^∗∗^		^∗^				
112	Mean	235.6	1.89	7.72	1.74	0.72	0.51	0.30	0.64	0.097	0.075
(*n* = 10)	SD	11.88	0.10	1.30	0.11	0.07	0.08	0.08	0.15	0.019	0.010
	SS			^∗∗^	^∗∗^		^∗^				
150	Mean	244.4	1.90	8.23	1.73	0.75	0.48	0.25	0.58	0.118	0.074
(*n* = 10)	SD	14.68	0.10	0.74	0.11	0.05	0.06	0.03	0.10	0.020	0.008
	SS			^∗∗^	^∗∗^			^∗∗^		^∗^	
187	Mean	247.2	1.95	8.92	1.82	0.76	0.49	0.30	0.62	0.125	0.074
(*n* = 10)	SD	13.72	0.08	1.11	0.11	0.06	0.06	0.09	0.12	0.03	0.01
	SS			^∗∗^	^∗∗^					^∗∗^	
225	Mean	231.6	1.83	8.81	1.71	0.71	0.44	0.22	0.53	0.126	0.067
(*n* = 10)	SD	15.88	0.10	1.05	0.20	0.07	0.05	0.04	0.10	0.03	0.01
	SS		^∗^	^∗∗^	^∗^			^∗∗^		^∗∗^	^∗∗^
Test for significance		NS	DN	DN	*U*	NS	DN	*U*	NS	DN	DN
Historical control range		208.0–297.0	1.83–2.17	5.18–8.53	1.36–2.34	0.63–0.85	0.32–0.56	0.18–0.47	0.42–1.11	0.07–0.14	0.063–0.104
*Recovery groups*											
*Male*									*Testes*	*Epididymides*	
0 (Control)	Mean	457.4	2.28	10.64	2.54	1.15	0.69	0.34	3.52	1.63	0.064
(*n* = 5)	SD	19.96	0.09	0.92	0.30	0.06	0.06	0.08	0.27	0.12	0.009
225	Mean	408.0	2.15	9.88	2.58	1.12	0.82	0.46	2.44	1.28	0.061
(*n* = 5)	SD	37.56	0.11	0.77	0.27	0.15	0.08	0.05	0.94	0.14	0.011
	SS	^∗^					^∗^	^∗^	^∗^	^∗∗^	
Test for significance		*T*	NS	NS	NS	NS	*T*	*T*	*T*	*T*	NS

*Female*									*Ovaries*	*Uterus*	
0 (Control)	Mean	244.0	2.08	5.98	1.55	0.74	0.47	0.25	0.63	0.092	0.071
(*n* = 5)	SD	15.56	0.12	0.41	0.20	0.07	0.04	0.07	0.16	0.015	0.008
225	Mean	235.0	1.96	6.72	1.61	0.75	0.49	0.35	0.76	0.096	0.075
(*n* = 5)	SD	17.54	0.07	1.04	0.16	0.05	0.07	0.05	0.13	0.012	0.013
	SS							^∗^			
Test for significance		NS	NS	NS	NS	NS	NS	*T*	NS	NS	NS

Abbreviations: DN, Duncan's multiple range test; NS, not Significant; SD, standard deviation; SS, statistically significant compared to control; *TT*-test versus control; *U*, Mann-Whitney *U*-test versus control. Remarks: Paired organs were weighed together. ^∗^*p* < 0.05, ^∗∗^*p* < 0.01.

**Table 9:** Percent organ weights relative to body weight in the 90-day study.

Group (mg/kg bw/day)		Brain	Liver	Kidneys	Heart	Spleen	Thymus	Testes	Epididymides	Adrenal glands
*Main study groups *(*n* = 10)										
*Male*										
0 (Control)	Mean	0.531	2.389	0.556	0.260	0.162	0.099	0.876	0.366	0.0167
(*n* = 10)	SD	0.034	0.156	0.033	0.018	0.023	0.018	0.065	0.031	0.0014
75	Mean	0.519	2.626	0.611	0.253	0.171	0.086	0.901	0.354	0.0174
(*n* = 10)	SD	0.038	0.067	0.077	0.019	0.010	0.010	0.070	0.061	0.0022
	SS		^∗∗^	^∗^			^∗^			
112	Mean	0.526	2.703	0.628	0.251	0.166	0.072	0.865	0.337	0.0165
(*n* = 10)	SD	0.020	0.173	0.064	0.019	0.020	0.011	0.075	0.051	0.0029
	SS		^∗∗^	^∗∗^			^∗∗^			
150	Mean	0.548	2.756	0.659	0.254	0.156	0.070	0.865	0.326	0.0181
(*n* = 10)	SD	0.036	0.167	0.028	0.014	0.021	0.014	0.111	0.033	0.0034
	SS		^∗∗^	^∗∗^			^∗∗^			
187	Mean	0.564	2.890	0.695	0.256	0.166	0.071	0.898	0.329	0.0187
(*n* = 10)	SD	0.037	0.203	0.070	0.026	0.021	0.013	0.077	0.034	0.0026
	SS		^∗∗^	^∗∗^			^∗∗^			
225	Mean	0.642	3.041	0.760	0.269	0.169	0.070	0.625	0.332	0.0212
(*n* = 10)	SD	0.050	0.173	0.060	0.014	0.018	0.012	0.270	0.060	0.0038
	SS	^∗∗^	^∗∗^	^∗∗^			^∗∗^			^∗∗^
Test for significance		DN	DN	*U*	NS	NS	DN	NS	NS	DN
Historical control range		0.403–0.606	2.055–3.156	0.452–0.634	0.211–0.284	0.119–0.194	0.063–0.129	0.642–0.963	0.279–0.424	0.009–0.020

*Female*								*Ovaries*	*Uterus*	
0 (Control)	Mean	0.817	2.519	0.642	0.298	0.189	0.139	0.261	0.0387	0.0343
(*n* = 10)	SD	0.039	0.152	0.022	0.020	0.022	0.018	0.054	0.0081	0.0046
75	Mean	0.821	3.055	0.701	0.302	0.214	0.121	0.294	0.0418	0.0327
(*n* = 10)	SD	0.038	0.292	0.026	0.024	0.020	0.026	0.060	0.0079	0.0026
	SS		^∗∗^	^∗∗^		^∗^				
112	Mean	0.804	3.270	0.738	0.305	0.216	0.125	0.270	0.0413	0.0318
(*n* = 10)	SD	0.062	0.478	0.038	0.017	0.031	0.030	0.056	0.0076	0.0047
	SS		^∗∗^	^∗∗^		^∗^				
150	Mean	0.781	3.373	0.707	0.306	0.198	0.101	0.237	0.0482	0.0304
(*n* = 10)	SD	0.059	0.284	0.038	0.023	0.029	0.016	0.043	0.0083	0.0032
	SS		^∗∗^	^∗∗^			^∗∗^			
187	Mean	0.789	3.607	0.736	0.307	0.199	0.122	0.251	0.0505	0.0297
(*n* = 10)	SD	0.04	0.39	0.04	0.02	0.02	0.03	0.04	0.01	0.00
	SS		^∗∗^	^∗∗^					^∗^	^∗^
225	Mean	0.794	3.808	0.736	0.307	0.191	0.096	0.226	0.0549	0.0291
(*n* = 10)	SD	0.05	0.45	0.06	0.02	0.02	0.01	0.04	0.01	0.01
	SS		^∗∗^	^∗∗^			^∗∗^		^∗∗^	^∗^
Test for significance		NS	*U*	DN	NS	DN	*U*	NS	DN	DN
Historical control range		0.731–1.000	2.183–3.189	0.508–0.951	0.236–0.333	0.139–0.227	0.078–0.169	0.161–0.465	0.029–0.054	0.025–0.045
*Recovery groups*										

*Male*								*Testes*	*Epididymides*	
0 (Control)	Mean	0.498	2.326	0.556	0.251	0.151	0.074	0.772	0.357	0.014
(*n* = 5)	SD	0.030	0.170	0.061	0.011	0.017	0.015	0.067	0.028	0.002
225	Mean	0.530	2.426	0.632	0.275	0.202	0.114	0.599	0.314	0.015
(*n* = 5)	SD	0.047	0.109	0.029	0.016	0.013	0.012	0.219	0.015	0.002
	SS			^∗^	^∗^	^∗∗^	^∗∗^		^∗^	
Test for significance		NS	NS	*T*	*T*	*T*	*T*	NS	*T*	NS

*Female*								*Ovaries*	*Uterus*	
0 (Control)	Mean	0.855	2.454	0.636	0.304	0.194	0.103	0.255	0.0376	0.0292
(*n* = 5)	SD	0.067	0.099	0.093	0.025	0.020	0.024	0.052	0.0055	0.0037
225	Mean	0.835	2.859	0.683	0.320	0.208	0.152	0.321	0.0415	0.0319
(*n* = 5)	SD	0.049	0.355	0.032	0.009	0.031	0.029	0.045	0.0079	0.0042
	SS		^∗^				^∗^			
Test for significance		NS	*T*	NS	NS	NS	*T*	NS	NS	NS

Abbreviations: DN, Duncan's multiple range test; NS, not significant; SD, standard deviation; SS, statistically significant compared to control; *TT*-test versus control; *U*, Mann-Whitney *U*-test versus control. Remarks: Paired organs were weighed together. ^∗^*p* < 0.05, ^∗∗^*p* < 0.01.

**Table 10:** Percent organ weights and body weight relative to brain weight in the 90-day study.

Group (mg/kg bw/day)		Body weight	Liver	Kidneys	Heart	Spleen	Thymus	Testes	Epididymides	Adrenal glands
*Main study groups *(*n* = 10)										
*Male*										
0 (Control)	Mean	18884.1	450.56	104.77	48.88	30.62	18.64	164.97	68.88	3.14
(*n* = 10)	SD	1185.15	34.64	5.10	2.36	5.06	2.84	7.92	3.63	0.28
75	Mean	19369.0	508.54	117.96	49.00	33.26	16.67	173.92	68.01	3.38
(*n* = 10)	SD	1497.30	40.18	14.45	4.07	3.92	1.79	12.27	9.21	0.50
	SS		^∗∗^	^∗^						
112	Mean	19038.2	514.06	119.31	47.83	31.62	13.78	164.49	64.17	3.12
(*n* = 10)	SD	740.19	29.38	11.02	3.88	3.40	2.31	12.18	9.98	0.49
	SS		^∗∗^	^∗∗^			^∗∗^			
150	Mean	18316.5	505.15	120.72	46.38	28.61	12.70	158.00	59.71	3.29
(*n* = 10)	SD	1184.75	49.68	10.54	3.01	4.43	1.94	20.19	7.31	0.65
	SS		^∗∗^	^∗∗^			^∗∗^		^∗∗^	
187	Mean	17807.0	513.9	123.7	45.6	29.5	12.8	159.3	58.4	3.3
(*n* = 10)	SD	1140.80	40.86	14.54	4.61	3.66	2.71	9.79	6.41	0.56
	SS		^∗∗^	^∗∗^			^∗∗^		^∗∗^	
225	Mean	15657.1	476.4	118.7	42.1	26.5	10.9	98.8	52.3	3.3
(*n* = 10)	SD	1229.16	49.52	8.88	3.80	4.43	1.90	47.54	12.61	0.60
	SS	^∗∗^		^∗^	^∗∗^	^∗^	^∗∗^	^∗∗^	^∗∗^	
Test for significance		DN	DN	DN	DN	DN	DN	*U*	*U*	NS
Historical control range		16500.0–24796.4	375.45–660.63	88.64–114.34	42.73–61.99	22.47–41.15	11.31–26.29	146.64–196.02	55.16–85.65	1.95–4.27

*Female*								*Ovaries*	*Uterus*	
0 (Control)	Mean	12265.9	309.18	78.80	36.44	23.16	17.14	31.91	4.74	4.20
(*n* = 10)	SD	607.51	26.15	5.43	2.16	2.60	2.68	6.16	0.94	0.59
75	Mean	12201.0	372.63	85.54	36.88	26.01	14.79	35.59	5.11	3.99
(*n* = 10)	SD	575.83	38.75	4.72	3.55	2.10	3.32	6.07	0.99	0.39
	SS		^∗∗^	^∗^						
112	Mean	12500.9	410.71	92.29	38.18	27.03	15.66	33.91	5.17	3.98
(*n* = 10)	SD	979.47	81.72	8.77	4.43	4.97	4.22	7.88	1.16	0.65
	SS		^∗∗^	^∗∗^						
150	Mean	12877.3	434.48	90.82	39.24	25.47	12.98	30.44	6.20	3.91
(*n* = 10)	SD	987.87	51.27	6.19	2.98	3.65	1.95	4.90	1.09	0.50
	SS		^∗∗^	^∗∗^			^∗∗^		^∗^	
187	Mean	12704.5	457.84	93.31	38.90	25.31	15.52	32.06	6.39	3.78
(*n* = 10)	SD	663.31	50.35	5.06	3.02	2.47	4.10	6.48	1.57	0.51
	SS		^∗∗^	^∗∗^					^∗∗^	
225	Mean	12648.4	481.25	93.02	38.73	24.04	12.23	28.71	6.90	3.66
(*n* = 10)	SD	894.76	60.74	9.13	2.63	2.32	1.99	5.83	1.42	0.56
	SS		^∗∗^	^∗∗^			^∗∗^		^∗∗^	
Test for significance		NS	*U*	DN	NS	NS	DN	NS	DN	NS
Historical control range		10000.0–13686.6	263.82–408.74	66.67–125.81	30.29–44.09	17.11–28.87	8.96–21.86	20.85–51.63	3.49–7.00	2.99–5.56
*Recovery groups*										
*Male*								*Testes*	*Epididymides*	
0 (Control)	Mean	20125.0	467.52	111.63	50.50	30.20	15.00	154.83	71.65	2.79
(*n* = 5)	SD	1271.17	35.97	11.76	3.41	1.85	3.21	9.87	4.90	0.30
225	Mean	18964.2	460.18	119.63	52.16	38.20	21.44	114.56	59.61	2.84
(*n* = 5)	SD	1517.80	43.82	8.62	5.60	2.96	1.57	46.71	5.88	0.45
	SS					^∗∗^	^∗∗^		^∗∗^	
Test for significance		NS	NS	NS	NS	*T*	*T*	NS	*T*	NS

*Female*								*Ovaries*	*Uterus*	
0 (Control)	Mean	11754.7	288.38	74.77	35.70	22.71	12.19	30.08	4.40	3.40
(*n* = 5)	SD	851.77	23.84	12.02	4.25	1.70	3.26	6.83	0.53	0.22
225	Mean	12010.5	342.70	82.02	38.43	24.95	18.16	38.57	4.94	3.83
(*n* = 5)	SD	731.38	41.11	6.49	1.42	3.32	3.03	5.94	0.68	0.55
	SS		^∗^				^∗^			
Test for significance		NS	*T*	NS	NS	NS	*T*	NS	NS	NS

Abbreviations: DN, Duncan's multiple range test; NS, not significant; SD, standard deviation; SS, statistically significant compared to control; *TT*-test versus control; *U*, Mann-Whitney *U*-test versus control.

Remarks: Paired organs were weighed together.

^∗^ *p* < 0.05^∗∗^*p* < 0.01.

**Table 11:** Summary of histopathology findings in the 90-day study.

Organs	Dose group (mg/kg bw/day)	Control (0)		75	112	150	187	225	
		Main group	Recovery group					Main group	Recovery group
	Observations								
*Male*		(*n* = 10)	(*n* = 5)	N/A	N/A	N/A	N/A	(*n* = 10)	(*n* = 5)
	Animals with no microscopic findings	7/10	4/5	N/A	N/A	N/A	N/A	4/10	2/5
Epididymides:	Lack of mature spermatozoa	0/10	0/5	0/10	0/10	0/10	0/10	6/10	3/5
Kidneys:	Pelvic dilatation, slight, one or two sides	1/10	0/5	1/1	2/2	2/2	1/1	2/10	0/5
	Chronic progessive nephropathy	0/10	0/5	1^3^/1	0/2	0/2	0/1	0/10	0/5
Lungs:	Alveolar emphysema	1^1^/10	0/5	/	/	/	/	0/10	0/5
	Hyperplasia of BALT	1^2^/10	1^2^/5	/	/	/	/	1^1^/10	0/5
Testes:	Decreased intensity of spermatogenesis	0/10	0/5	0/10	0/10	0/10	0/10	6^3–4^/10	3^3–4^/5
*Female*		(*n* = 10)	(*n* = 5)	N/A	N/A	N/A	N/A	(*n* = 10)	(*n* = 5)
	Animals with no microscopic findings	7/10	0/5	N/A	N/A	N/A	N/A	6/10	1/5
Kidneys:	Pelvic dilatation, slight, one or two sides	0/10	1/5	2/2	1/1	/	1/1	1/10	0/5
Liver:	Congestion	0/10	0/5	/	/	/	2/2	1/10	0/5
	Fibrosis in the Glisson's capsule	0/10	2^2^/5	/	/	/	/	0/10	0/5
Lungs:	Alveolar emphysema, minimal	1^1^/10	0/5	/	/	/	0/1	0/10	0/5
	Acute hemorrhage, mild	0/10	1^1^/5	/	/	/	1^3^/1	0/10	0/5
	Hyperplasia of BALT, minimal	0/10	0/5	/	/	/	0/1	0/10	1^2^/5
Thymus:	Acute hemorrhage, mild	0/10	0/5	1^2^/1	/	/	/	0/10	0/5
Uterus:	Dilatation	2/10	2/5	/	/	/	/	2/10	3/5

Abbreviations: /, not examined; BALT, bronchus associated lymphoid tissue; N/A, not applicable (only read-down organs/tissues and gross lesions were examined). Data represent incidence of the observation (number of animals with observation per number of animals examined). Organs without lesions in 15/15 control and high-dose animals or gross lesions in the lower dose groups not shown. Superscripts represent grade of lesion: 1 = minimal, 2 = mild, 3 = moderate, 4 = severe.

## Data Availability

The mean data sets generated and utilized for statistical analysis to support the findings of these studies are included within the article or in the supplementary information files. All other raw and processed data used to support the findings of these studies are available from the corresponding author upon request.

## Supplementary Materials

Supplementary Materials In order that all mean data sets subject to statistical analysis and upon which the authors drew interpretations and conclusions are available to readers, tables for body weight gain, food consumption, feed efficiency, and absolute and relative organ weights were provided as supplementary material.
