# Supplementary material for: A Toxicological Evaluation of Methylliberine (Dynamine®)
Source: J Toxicol. 2019 Oct 27;2019:4981420. doi: 10.1155/2019/4981420 (PMC6930730; doi:10.1155/2019/4981420)
Supplement: Supplementary Materials — In order that all mean data sets subject to statistical analysis and upon which the authors drew interpretations and conclusions are available to readers, tables for body weight gain, food consumption, feed efficiency, and absolute and relative organ weights were provided as supplementary material. [file 4981420.f1.pdf]

Table S1. Food Consumption in the 90-day Study

Group (mg/kg bw/day)		Daily mean food consumption (g/animal/day)																
		**Treatment Period**													**Recovery Period**			
	**Days**	**0-7**	**7-14**	**14-21**	**21-28**	**28-35**	**35-42**	**42-49**	**49-56**	**56-63**	**63-70**	**70-77**	**77-84**	**84-89**	**90-97**	**97-104**	**104-111**	**111-117**
	**Weeks**	**1**	**2**	**3**	**4**	**5**	**6**	**7**	**8**	**9**	**10**	**11**	**12**	**13**	**R1**	**R2**	**R3**	**R4**
**Male**																		
0 (Control)	Mean	23.7	25.1	24.0	24.2	23.6	22.1	22.7	22.5	23.1	22.4	21.7	23.6	23.2	24.6	25.3	23.8	20.8
(n = 15†)	SD	1.58	1.68	1.68	1.67	1.52	1.47	1.71	1.70	1.61	1.58	1.53	1.56	1.83	1.51	2.27	1.42	1.43
75	Mean	22.9	24.2	23.1	23.3	23.5	22.4	23.6	23.0	23.6	23.3	22.6	24.6	24.7				
(n = 10)	SD	1.42	1.52	1.85	2.04	1.88	1.78	2.48	2.46	2.30	2.63	2.47	2.53	2.64				
112	Mean	22.9	24.5	23.8	23.9	23.3	22.1	23.1	23.0	23.3	23.0	22.0	24.4	24.6				
(n = 10)	SD	1.87	2.10	1.23	1.39	1.23	1.44	1.24	1.50	1.22	1.38	1.48	1.38	1.17				
150	Mean	21.7	23.5	22.5	23.0	23.0	22.2	22.8	22.3	22.7	22.1	21.6	23.4	23.5				
(n = 10)	SD	1.77	2.25	1.98	1.46	1.66	1.95	2.16	2.26	2.39	2.42	2.50	2.69	2.44				
	SS	**	*															
187	Mean	21.0	22.4	21.7	23.1	23.0	22.5	22.0	21.8	22.3	23.0	21.7	23.0	23.9				
(n = 10)	SD	1.03	1.30	2.14	1.56	1.47	1.39	1.56	1.69	1.91	2.21	1.52	1.20	1.59				
	SS	**	**	**														
225	Mean	20.5	22.3	20.9	21.9	21.9	20.4	21.6	20.9	21.4	21.0	20.6	22.1	22.5	23.5	24.9	24.1	20.2
(n = 15†)	SD	1.35	1.30	1.58	1.53	1.99	1.66	1.87	1.82	2.48	2.18	2.08	2.41	2.81	1.85	2.81	2.38	2.33
	SS	**	**	**	**	*	**		*	*								
Test for Significance		DN	DN	DN	DN	DN	DN	NS	DN	DN	NS	NS	NS	NS	NS	NS	NS	NS
**Female**																		
0 (Control)	Mean	17.2	18.0	16.9	17.2	16.8	16.0	16.6	16.4	17.0	16.6	15.8	17.7	17.8	17.4	18.1	16.9	14.5
(n = 15†)	SD	1.16	1.25	1.07	1.20	1.26	1.41	0.99	1.12	1.47	1.39	1.44	1.70	1.63	1.09	0.88	0.95	0.99
75	Mean	15.9	17.4	16.3	16.5	16.5	15.6	16.9	16.5	17.3	17.1	16.2	18.2	18.6				
(n = 10)	SD	0.82	1.36	1.17	1.00	1.17	1.14	1.31	0.78	0.96	1.27	1.21	0.77	0.70				
	SS	**												*				
112	Mean	15.2	17.1	15.9	16.4	16.3	15.7	17.0	16.6	17.0	17.1	16.0	18.3	18.7				
(n = 10)	SD	1.11	1.43	1.44	1.23	1.14	1.24	1.18	1.23	1.34	1.49	1.25	1.74	1.72				
	SS	**																
150	Mean	14.9	17.4	17.1	17.4	17.4	17.8	18.4	17.6	17.6	17.5	16.6	18.7	18.8				
(n = 10)	SD	1.20	1.82	2.16	2.38	2.14	3.28	2.61	1.90	1.79	1.48	1.44	1.47	1.16				
	SS	**																
187	Mean	15.1	17.8	16.8	17.5	17.4	17.2	17.1	17.0	17.8	17.5	17.0	18.8	19.2				
(n = 10)	SD	0.69	0.69	1.13	1.55	1.55	2.00	0.76	1.20	0.91	1.37	1.05	1.31	1.25				
	SS	**												*				
225	Mean	14.4	17.2	16.4	16.9	17.0	16.6	17.0	17.1	17.2	17.0	16.1	17.9	17.8	18.1	18.2	17.3	14.5
(n = 15†)	SD	1.23	1.61	1.43	1.52	2.03	1.97	1.57	1.82	1.51	1.50	1.35	1.90	2.07	1.45	1.23	1.20	0.89
	SS	**																
Test for Significance		DN	NS	NS	NS	NS	NS	NS	NS	NS	NS	NS	NS	U	NS	NS	NS	NS

Abbreviations: DN, Duncan's multiple range test; NS, Not Significant; SD, standard deviation; SS, statistically significant compared to control; U, Mann-Whitney U-test versus control.

†Recovery Period (n = 5)

*p < 0.05; **p < 0.01

Table S2. Feed Efficiency in the 90-day Study

Group		Feed efficiency (g food/ g bwg)																		
		Treatment Period														Recovery Period				
	Days	0-7	7-14	14-21	21-28	28-35	35-42	42-49	49-56	56-63	63-70	70-77	77-84	84-89	0-89	90-97	97-104	104-111	111-117	90-117
	Weeks	1	2	3	4	5	6	7	8	9	10	11	12	13	1-13	R1	R2	R3	R4	R1-4
**Male**																				
0 (Control)	Mean	3.72	4.59	5.84	6.85	8.31	10.52	13.69	11.99	18.94	23.95	15.24	40.44	57.34	9.13	18.73	50.51	72.13	14.40	24.01
(mg/kg bw/day)	SD	0.33	0.47	0.84	0.99	1.79	2.32	10.23	3.54	8.29	11.05	4.66	43.07	38.52	1.85	4.47	21.69	64.44	1.70	3.52
	n	15	15	15	15	15	15	15	15	15	14	15	15	11	15	5	5	4	5	5
75	Mean	4.00	5.43	6.83	6.64	7.51	10.48	11.63	10.98	25.76	27.49	17.72	20.40	53.96	9.00					
(mg/kg bw/day)	SD	0.46	0.97	1.58	1.09	1.37	2.51	2.83	2.18	27.71	18.02	11.71	7.11	36.64	0.68					
	n	10	10	10	10	10	10	10	10	10	10	10	10	7	10					
	SS	*	*																	
112	Mean	4.09	4.94	5.49	6.82	7.65	11.75	12.03	13.68	23.57	44.22	16.69	33.26	60.20	8.94					
(mg/kg bw/day)	SD	0.49	0.63	0.69	1.39	1.17	6.56	2.63	6.74	19.39	43.24	7.34	49.13	43.29	0.86					
	n	10	10	10	10	10	10	10	10	10	10	10	10	4	10					
	SS	*																		
150	Mean	4.32	5.09	7.36	8.85	7.86	11.86	16.14	12.97	18.20	65.56	17.31	36.30	38.63	9.79					
(mg/kg bw/day)	SD	0.65	0.69	2.76	4.14	1.59	2.38	5.99	2.89	7.48	58.13	6.78	31.43	30.92	0.68					
	n	10	10	10	10	10	10	10	10	10	10	10	9	8	10					
	SS	*													**					
187	Mean	4.46	6.50	7.01	7.55	10.07	12.53	23.29	12.99	19.72	35.72	28.33	22.29	36.29	10.58					
(mg/kg bw/day)	SD	0.67	1.78	1.95	1.83	6.98	3.61	7.72	5.26	8.13	47.08	19.07	5.49	21.56	1.14					
	n	10	10	10	10	10	10	9	10	10	8	10	8	8	10					
	SS	**	**					**							**					
225	Mean	5.04	7.61	8.62	8.66	10.17	23.99	22.37	19.31	43.46	29.36	17.71	30.82	47.69	12.33	15.26	7.94	13.41	18.23	10.84
(mg/kg bw/day)	SD	0.89	2.14	2.29	2.34	2.80	16.95	12.81	10.91	40.67	19.42	4.38	30.89	26.25	1.36	11.80	1.98	8.14	11.11	3.32
	n	15	15	15	15	15	15	14	15	13	14	14	14	10	15	5	5	5	5	5
	SS	**	**	**	**	*	**	**							**		**			**
Test for Significance		U	U	U	U	U	U	U	NS	NS	NS	NS	NS	NS	U	NS	T	NS	NS	T
**Female**																				
0 (Control)	Mean	6.23	7.59	9.17	11.81	18.46	17.81	36.24	28.12	22.56	32.08	71.72	30.31	44.00	14.06	49.00	31.39	89.00	35.40	77.91
(mg/kg bw/day)	SD	0.98	2.20	5.80	3.84	9.26	11.30	37.56	9.14	12.84	16.85	41.15	28.07	36.01	1.51	14.05	26.21	46.67	43.59	23.09
	n	15	15	15	15	15	15	14	11	15	11	8	12	12	15	3	3	2	4	4
75	Mean	5.91	6.96	9.30	14.11	10.39	32.52	19.89	35.48	16.60	39.63	42.06	32.38	34.78	13.38					
(mg/kg bw/day)	SD	1.50	1.32	2.74	7.80	2.87	39.86	10.97	35.39	4.43	33.85	35.69	22.36	32.89	1.36					
	n	10	10	10	10	10	10	9	9	10	8	6	7	9	10					
	SS					**														
112	Mean	5.28	7.24	9.19	12.03	12.07	15.42	30.32	30.26	33.65	52.14	36.61	39.54	27.99	13.24					
(mg/kg bw/day)	SD	1.41	1.02	2.83	3.62	4.47	5.13	36.65	38.30	31.67	45.85	11.64	10.80	25.64	1.17					
	n	10	10	10	10	10	10	10	9	10	10	6	9	10	10					
	SS					*														
150	Mean	5.10	6.92	10.16	12.67	10.45	23.11	28.87	16.63	28.83	51.03	24.05	41.19	61.38	13.74					
(mg/kg bw/day)	SD	0.72	0.85	2.83	5.51	2.75	14.44	32.65	4.81	7.92	37.00	7.31	32.72	38.94	1.64					
	n	10	10	10	10	10	10	10	10	8	9	9	9	8	10					
	SS	*				**			**	*										
187	Mean	5.07	6.87	10.01	10.68	12.45	15.16	38.07	21.73	22.80	36.23	24.93	59.05	40.68	13.21					
(mg/kg bw/day)	SD	0.95	2.13	1.85	2.09	3.05	3.91	31.88	12.44	8.78	31.57	17.74	47.70	29.43	1.36					
	n	10	10	10	10	10	10	10	10	10	8	8	10	10	10					
	SS	*				*														
225	Mean	5.62	8.67	10.21	11.68	13.85	22.89	28.52	15.92	42.45	37.70	43.77	31.92	29.66	14.41	-	16.81	46.56	61.33	143.41
(mg/kg bw/day)	SD	0.98	3.18	4.69	4.94	6.90	13.18	26.55	4.02	39.06	26.50	38.95	12.64	22.86	1.18	-	6.23	12.51	34.43	112.16
	n	15	15	15	15	15	14	13	15	15	14	10	12	11	15	-	3	3	3	2
	SS								**	*										
Test for Significance		DN	NS	NS	NS	U	NS	NS	U	U	NS	NS	NS	NS	NS	-	NS	NS	NS	NS

Abbreviations: bwg, body weight gain; DN, Duncan's multiple range test; NS, Not Significant; SD, standard deviation; SS, statistically significant compared to control; T, T-test versus control; U, Mann-Whitney U-test versus control.

-No data (bwg was negative for 5 of 5 animals during this time period)

*p < 0.05; **p < 0.01

Table S3. Summary of Gross Pathology Findings in the 90-day Study

	Dose Group (mg/kg bw/day)	Control (0)		75	112	150	187	225	
**Organs**	**Observations**	**Main Group (n=10)**	**Recovery Group (n=5)**	**(n=10)**	**(n=10)**	**(n=10)**	**(n=10)**	**Main Group (n=10)**	**Recovery Group (n=5)**
**Male**									
	No macroscopic findings	8/10	5/5	9/10	8/10	8/10	9/10	5/10	1/5
Kidneys:	Pyelectasia - one or both sides	1/10	0/5	1/10	2/10	2/10	1/10	2/10	1/5
Testes:	Smaller than normal	0/10	0/5	0/10	0/10	0/10	0/10	5/10	3/5
Skin:	Scar - left side shoulder	1/10	0/5	0/10	0/10	0/10	0/10	0/10	0/5
**Female**									
	No macroscopic findings	8/10	1/5	3/10	4/10	6/10	4/10	5/10	2/5
Lungs:	Hemorrhage	0/10	0/5	0/10	0/10	0/10	1/10	0/10	0/5
Liver:	Congestion	0/10	0/5	0/10	0/10	0/10	1/10	0/10	0/5
	Brown colored	0/10	0/5	0/10	0/10	0/10	1/10	1/10	0/5
Diaphragm:	*Hernia diaphragmatica*	0/10	2/5	0/10	0/10	0/10	0/10	0/10	0/5
Kidneys:	Pyelectasia - one or both sides	0/10	1/5	2/10	1/10	0/10	1/10	1/10	0/5
Uterus:	Hydrometra - slight, moderate or marked	2/10	2/5	4/10	5/10	4/10	5/10	2/10	3/5
Ovaries:	Larger than normal	0/10	0/5	0/10	0/10	0/10	1/10	2/10	0/5
Thymus:	Hemorrhage	0/10	0/5	1/10	0/10	0/10	0/10	0/10	0/5

Data represent incidence of the observation (number of animals with observation per number of animals examined).
Organs without lesions not shown.
